# Supplementary material for: Excess all-cause deaths stratified by sex and age in Peru: a time series analysis during the COVID-19 pandemic
Source: BMJ Open. 2022 Mar 10;12(3):e057056. doi: 10.1136/bmjopen-2021-057056 (PMC8914408; doi:10.1136/bmjopen-2021-057056)
Supplement: Author's manuscript [file bmjopen-2021-057056.draft_revisions.pdf]

BMJ Open is committed to open peer review. As part of this commitment we make the peer review history of every article we publish publicly available.

When an article is published we post the peer reviewers' comments and the authors' responses online. We also post the versions of the paper that were used during peer review. These are the versions that the peer review comments apply to.

The versions of the paper that follow are the versions that were submitted during the peer review process. They are not the versions of record or the final published versions. They should not be cited or distributed as the published version of this manuscript.

BMJ Open is an open access journal and the full, final, typeset and author-corrected version of record of the manuscript is available on our site with no access controls, subscription charges or pay-per-view fees (http://bmjopen.bmj.com).

If you have any questions on BMJ Open's open peer review process please email info.bmjopen@bmj.com

BMJ Open

Excess all-cause deaths stratified by sex and age during the COVID-19 pandemic in Peru: An analysis of national deaths

Journal:	*BMJ Open*
Manuscript ID	bmjopen-2021-057056
Article Type:	Original research
Date Submitted by the Author:	09-Sep-2021
Complete List of Authors:	Ramírez-Soto, Max Carlos; Universidad Peruana Cayetano Heredia; Universidad Tecnológica del Perú<br>Ortega-Cáceres, Gutia; Universidad Ricardo Palma<br>Arroyo-Hernández, Hugo; Instituto Nacional de Salud
Keywords:	COVID-19, Epidemiology < INFECTIOUS DISEASES, EPIDEMIOLOGY, PUBLIC HEALTH

SCHOLARONE™
Manuscripts

I, the Submitting Author has the right to grant and does grant on behalf of all authors of the Work (as defined in the below author licence), an exclusive licence and/or a non-exclusive licence for contributions from authors who are: i) UK Crown employees; ii) where BMJ has agreed a CC-BY licence shall apply, and/or iii) in accordance with the terms applicable for US Federal Government officers or employees acting as part of their official duties; on a worldwide, perpetual, irrevocable, royalty-free basis to BMJ Publishing Group Ltd ("BMJ") its licensees and where the relevant Journal is co-owned by BMJ to the co-owners of the Journal, to publish the Work in this journal and any other BMJ products and to exploit all rights, as set out in our licence.

The Submitting Author accepts and understands that any supply made under these terms is made by BMJ to the Submitting Author unless you are acting as an employee on behalf of your employer or a postgraduate student of an affiliated institution which is paying any applicable article publishing charge ("APC") for Open Access articles. Where the Submitting Author wishes to make the Work available on an Open Access basis (and intends to pay the relevant APC), the terms of reuse of such Open Access shall be governed by a Creative Commons licence – details of these licences and which Creative Commons licence will apply to this Work are set out in our licence referred to above.

Other than as permitted in any relevant BMJ Author's Self Archiving Policies, I confirm this Work has not been accepted for publication elsewhere, is not being considered for publication elsewhere and does not duplicate material already published. I confirm all authors consent to publication of this Work and authorise the granting of this licence.

For peer review only

Excess all-cause deaths stratified by sex and age during the COVID-19 pandemic in Peru: An analysis of national deaths

Max Carlos Ramírez-Soto [1,2,*], Gutia Ortega Cáceres [3], Hugo Arroyo-Hernández [4]

[1] School of Public Health and Administration, Universidad Peruana Cayetano Heredia, Lima, Peru

[2] Facultad de Ciencias de la Salud, Universidad Tecnológica del Perú, Lima, Peru

[2] Escuela de Posgrado, Universidad Ricardo Palma, Lima, Peru

[2] Oficina General de Información y Sistemas, Instituto Nacional de Salud, Lima, Peru

*** Correspondence:**

Max Carlos Ramírez-Soto;

School of Public Health and Administration, Universidad Peruana Cayetano Heredia, Lima, Peru

maxcrs22@gmail.com; Tel.: +51 984887238

Conflicts of Interest: The authors declare no conflict of interest.

Funding: Please add: "This research received no external funding"

Words: 2300

Figures: 06

For peer review only - http://bmjopen.bmj.com/site/about/guidelines.xhtml

Abstract

Background. During the COVID-19 pandemic, excess all-cause mortality has been reported in Peru, but it has not been documented how excess mortality varies across population subgroups. In this study, we estimated the excess all-causes deaths and excess death rates during the COVID-19 pandemic in 25 Peruvian regions, stratified by sex and age groups.

Methods. Using the official mortality data set released by the Sistema Informatico Nacional de Defunciones (SINADEF) at the Ministry of Health of Peru, we calculated the excess all-cause deaths and excess death rates. We compared the deaths observed in 2020 with the deaths expected (2017–2019) from epidemiological weeks 1 to 52. The odds ratios (OR) for excess all-cause mortality were estimated according to sex, age, and department and summarized with a random effects meta-analysis.

Results. In the period between January and December 2020, we estimated an excess of 68,608 (117%) deaths in men and 34,742 (69%) deaths in women, corresponding to an excess death rate of 411 per 100,000 men and 201 per 100,000 women compared to the expected mortality rates. The number of excess deaths increased with age and was higher in men aged 60–69 years (217%) compared with women (121%). Men between the ages of 40 and 79 years experienced twice the rate of excess deaths compared with the rate expected. In eight regions, the excess deaths were higher than 100% in men, and in seven departments the excess deaths were higher than 70% in women. Men in five regions and women in one region had two times increased odds of excess death than the expected mortality. There were differences in excess mortality according to the temporal distribution by epidemiological week.

Conclusion. Approximately one hundred thousand excess all-cause deaths occurred in 2020 in Peru. The age-stratified excess death rates were higher in men than in women. There were strong excesses in geographical and temporal mortality patterns according to region.

Keywords: excess mortality; sex; mortality rate; COVID-19; Peru

For peer review only - http://bmjopen.bmj.com/site/about/guidelines.xhtml

Strengths and limitations of this study

- While there have been numerous studies on excess all-cause deaths and COVID-19 in high-income countries, there are gaps in the epidemiological data on excess deaths in many low- and middle-income countries of Latin America, including Peru.
- Males had a greater number of excess deaths and higher excess death rates (per 100,000) than females across all age groups. Men and women in the 60–69 years age group had two times increased odds of excess mortality in 2020 compared to the expected mortality.
- There were strong excesses in geographical and temporal mortality patterns, and some regions showed staggering increases to rates of 500% in men.
- The main strength of our study was the large number of deaths included (127,000 all-cause deaths) for estimating the excess mortality.
- We present the results of a retrospective analysis and so we are not able to comment on causality in COVID-19 mortality.

For peer review only

For peer review only - http://bmjopen.bmj.com/site/about/guidelines.xhtml

Introduction

Following the outbreak of COVID-19 in China in 2019, SARS-CoV-2 spread rapidly worldwide.[1] During the following weeks, there was a rapidly increase in the incidence and mortality rates of COVID-19 in European countries, the USA, and Latin American countries.[2] Previous studies on excess deaths (the gap between observed and expected deaths) during the COVID-19 pandemic have found that COVID-19-related deaths and deaths from other diseases increased along with age and were higher in men 60 years of age and older compared with women.[3-10] More recently, the assessment of the direct and indirect effects of the COVID-19 pandemic on mortality has revealed that approximately one million excess deaths occurred in 2020 in 29 high-income countries. Additionally, these excess death rates were higher in men than in women in almost all countries.[11]

Peru is one of the countries that has been worst affected by the COVID-19 pandemic. The first cases in Peru were reported in March 2020. During the following weeks, rapidly increasing numbers of cases and fatalities were observed across many Peru departments. By the end of December 2020, about 1.0 million confirmed COVID-19 cases and more than 90,000 deaths had been officially reported.[12] In Peru, data on excess all-cause deaths have been reported, but analyses have not accounted for age and sex standardization. From 29 March 2020 (week 14), a significant rate of excess deaths in comparison with the expected deaths based on the figures from 2017 to 2019 has been observed at the national level, with this rate reaching the highest level in week 32 and then decreasing.[13] This increase in all-cause deaths is concomitant with the COVID-19 pandemic, as it has previously been shown that COVID-19 mortality rates increase with age and are high in men aged 60 years and over.[14,15] Despite these findings, to date, the excess mortality stratified by sex, age, and region is not known at the national level. The assessment of the impact of the COVID-19 pandemic on mortality in Peru should include the analysis of both the direct effect of the pandemic on deaths caused by COVID-19 and the indirect effect of the pandemic on deaths unrelated to COVID-19, since patients with chronic conditions may have been turned away from the health care system because of concerns relating to COVID-19 infection and social and economic changes.

In this study, we estimate the excess all-cause deaths and the excess death rate during the first year of the COVID-19 pandemic in 25 Peruvian regions, stratified by sex, age, group, and epidemiological week.

Methods

Data collection

We performed an observational retrospective study using weekly death data from the Sistema Informatico Nacional de Defunciones (SINADEF) at the Ministry of Health of Peru.[16] We used deaths registers for 25 Peruvian regions from the 01 January through to 31 December 2020 (1–52 epidemiological weeks) and the preceding 3 years (2017–2019).

SINADEF is a computer application. It registers data on deaths, generates death certificates, and creates a statistical report; it includes fetal deaths and the deaths of unidentified persons.[16] Open access data includes information stratified by age/sex, the basic cause of death, the year, the International Classification of Diseases 10th Revision (ICD-10), and the geographic distribution. Other information is not available in SINADEF. All-cause deaths where the underlying cause was known were available, with information on region, age group (0–29, 30–39, 40–49, 50–59, 60–69, 70–79, and 80 and over), and sex included. Records where the place of residence, age, sex, or year was missing were excluded. Underlying cause of death

For peer review only - http://bmjopen.bmj.com/site/about/guidelines.xhtml

was obtained from the death certificate and by using ICD-10. The underlying cause of death is defined by the WHO as the disease or injury that initiated the series of events that led directly to death, or the circumstances of the accident or violence that produced the fatal injury. This condition may be the main reason for or a reason contributing to the event of death.

Patient and public involvement

We used publicly available death statistics, therefore, there was no direct patient or public involvement.

Ethics statements

This study does not involve human participants. Weekly death data were obtained of SINADEF. In SINADEF, all open access data are fully anonymous and are published as part of routine surveillance (https://www.datosabiertos.gob.pe/dataset/informaci%C3%B3n-de-fallecidos-del-sistema-inform%C3%A1tico-nacional-de-defunciones-sinadef-ministerio). Therefore, this study was exempt from review by an ethics board.

Excess mortality analysis

We estimated the excess deaths and the excess death rates according to sex and age, as well as in each region by sex. The excess death rate was calculated using the difference between the deaths observed in 2020 and the deaths expected based on data from the years 2017–2019. The proportion of excess deaths was computed as [(Observed deaths/Expected deaths)-1]*100%. Mortality rates per 100,000 inhabitants for both sexes were also calculated by dividing the number of deaths per region, standardized by the estimated population of each region. We calculated the observed mortality rate for the years 2017 to 2019 and the mortality rate expected in 2020 for both sexes according to age and region. The excess death rate was calculated using the difference between the mortality rate observed in 2020 and the mortality rate expected based on the years 2017–2019. Population counts used for calculating the mortality rates were obtained from INEI of Peru.[16] In addition, odds ratios (ORs) with 95% confidence intervals (CIs) for mortality observed vs. mortality expected according to sex were estimated separately for each age stratum and region. These results were summarized in a random-effects meta-analysis with individual reports weighted using the weights indicated. The Mantel–Haenszel methods were used to calculate the random effects estimates. $P<0.05$ was considered statistically significant. The meta-analysis was performed in RevMan 5, Cochrane.

Results

Excess death and excess death rates by sex and age

Between January 1, 2020, and December 31, 2020, a total of 127,000 all-cause deaths (MR 784 deaths per 100,000 men) were reported in men. In comparison with the expected 58,392 deaths (MR 373 per 100,000 men), the number of excess deaths during the same period was 68,608 (117%), and the excess death rate was 411 deaths per 100,000 men. During the same period, a total of 85,240 (MR, 519 deaths per 100,000 men) all-cause deaths were reported in women. In comparison with the expected 50,498 deaths (MR, 317 deaths per 100,000 women), the number of excess deaths during the same period was 34,742 (69%), and the excess death rate was 201 deaths per 100,000 women.

The number of excess deaths increased with age and was higher in men aged 60–69 years (217%) compared with women (121%) (Figure 1A). The all-cause mortality rate in relation to the expected death rate increased with age and was higher in individuals aged 80 years or more in both sexes (Figure 1B). In addition, the increase in the all-cause mortality rate was more than twice as high in men compared with that in women.

For peer review only - http://bmjopen.bmj.com/site/about/guidelines.xhtml

ORs for excess death rates stratified by sex and age

In Figure 2, our meta-analysis shows a continuous age-dependent increase in the number of excess deaths in men and women. Overall, men and women had 1.92 (95% CI, 1.49–2.48) and 1.57 (95% CI, 1.33–1.84) times increased odds of excess mortality compared to the expected mortality, respectively. Men in the 40–79 years age group had a two times higher odds of excess mortality. Men in the 60–69 years age group had a 2.95 (95% CI, 2.87-3.02) times increased odds of excess mortality, while women had a 2.05 (95% CI, 1.99-2.11) times increased odds (Figure 2A,B).

Excess death and excess death rates by sex and regions

The excess all-cause death rate relative to the expected death rate varied between regions. For men, the excess death rate varied from 34% in Cusco to 199% in Lima, while for women, it varied from 7% in Tacna and 125% in Lima. The five regions with the highest excess death rates in men were Lima (199%), Callao (143%), Piura (137%), Moquegua (129%), and Arequipa (123%); the five regions with the highest excess deaths in women were Lima (125%), Callao (94%), Paso (91%), Tumbes (83%), and Piura (78%) (Figure 3A). The highest excess death rates in men (per 100,000 men) and women (per 100,000 women) occurred in Lima, Callao, Moquegua, Arequipa, Ica, and Tumbes (Figure 3B).

ORs for excess death rates stratified by sex and regions

In Figure 4, our meta-analysis shows a continuous increase in the rate of excess deaths in men and women. Men in Lima, Callao, Piura, Arequipa, Loreto, Tumbes, and Moquegua had two times increased odds of excess mortality compared to the expected mortality (Figure 4A), while only in Lima did women have a 2.16 (95% CI, 2.12–2.21) times increased odds of excess mortality compared to the expected mortality (Figure 4B).

Temporal distribution of excess deaths by sex and region

Figure 5 shows that the rates of excess deaths increased at the beginning of March and reached a peak at the end of the months of May and August. Some regions showed staggering increases, with rates of excess deaths reaching 500% in men in Loreto in week 18. The rates of excess deaths decreased by week 52 (Figure 6).

Discussion

During the COVID-19 pandemic. Peru experienced one of the highest excess all-cause mortality rates in the world, along with Bulgaria, North Macedonia, and Serbia.[17] Excess all-cause mortality is recognized as a robust and comparable indicator of mortality associated with the COVID-19 pandemic.[18] Some countries have reported that approximately two thirds of their excess deaths were reported as COVID-19-related deaths.[19] However, in countries such as Peru, where the effects of the pandemic have exceeded the reporting systems for mortality related to COVID-19, the measurement of excess mortality can help guide public health decisions and actions.

For peer review only - http://bmjopen.bmj.com/site/about/guidelines.xhtml

According to our analysis, men in Peru had an excess mortality rate almost double that of women, This differs from the data reported in most industrialized and high-income countries where no great difference is evident. For example, the excess mortality in Peruvian men (117%) was double that reported for England and Wales (63%), which, together with Spain, were the countries that experienced the highest excess mortality in Europe (18); this is also higher than the rate reported in other countries of the American continent, such as Mexico (51%) and Brazil (61%).[8,21] Excess mortality in men is likely related to the COVID-19 pandemic, as SARS-CoV-2 has a greater transmissibility in men, which is due mainly to their social and cultural behaviors. It has also been proposed that women have a better protective factor immune response due to their higher number of B lymphocytes and the higher intensity of their inflammatory response against viral diseases.[22,23]

As in Peru, other countries have also shown a reduction in mortality, or avoided mortality, in younger age groups.[24] This due to the lower number of unintentional injuries occurring due to COVID-19 containment measures, such as occupational or automobile accidents, the latter being the main cause of years of life lost due to premature death.[25] However, excess mortality occurred in a wider range of age groups for men (40–79 years old); this may be related to the high prevalence of chronic diseases in adult men, which increases the likelihood of death due to COVID-19.[26,27]

The excess mortality seen in Peru had an asynchronous temporal and geographical distribution. However, men were found to be twice as likely to die in more regions than women were. Regions along the coast (Lima, Callao, Piura, Tumbes), in the Andes (Arequipa, Moquegua), and in the Amazon (Loreto) have large cities with high levels of commercial, migratory, and tourist exchange. In contrast, the regions of the Andes located at higher altitudes had lower mortality rates. This has been related to the physiological adaptation of this population to a hypoxic environment, which may have protected them from severe impacts of the acute infection caused by SARS-CoV-2; in addition, these areas have a lower prevalence of diabetes, obesity, and hypertension.[28] Therefore, the excess all-cause deaths are likely to be a direct effect of the COVID-19 pandemic. These characteristics, together with the low compliance with social distancing measures, the closure of economic activities during the pandemic, the entry of new variants of SARS-CoV-2 with a greater transmissibility, the difficulty of early diagnosis, the collapse of hospitals, and the shortage of medical oxygen could explain the higher excess mortality in Peru.[29,30] The changes in population (male and female) behavior brought about by lockdown measures could also have had effects on excess deaths and the mortality rate. Finally, during the country-wide lockdown implemented in Peru, access to healthcare was limited and many medical procedures were delayed. Therefore, the effects of the lockdown may be another explanation for the excess number of deaths and excess death rate.

Our study has several important limitations. First, although SINADEF is an official database, there may have been delays in its registration of deaths. Therefore, the number of excess all-cause deaths registered might have been underestimated. Second, despite the fact that there are complex models for calculating excess all-cause deaths, we used a simple model for our analysis. Despite this, our findings in numerical terms are similar to those of MINSA of Peru. Finally, although it would be ideal to wait until the COVID-19 pandemic is over to analyze the mortality data, there is a need for more studies to be carried out on excess deaths in men and women, so this study is warranted.

Conclusion

Approximately one hundred thousand excess deaths occurred in men and women during the COVID-19 pandemic in Peru. The age-stratified excess death rates were higher in men than in women, and men between the ages of 40 and 79 years experienced twice the rate of excess

For peer review only - http://bmjopen.bmj.com/site/about/guidelines.xhtml

deaths compared with the rate expected. Strong excesses in geographical and temporal mortality patterns by region were found in both men and women. These findings reveal the impact of the COVID-19 pandemic on all-cause mortality up to the point where vaccination against SARS-CoV- 2 started to become available in Peru.

Footnotes

Contributors. MCRS designed the study and oversaw the acquisition of data. MCRS, GOC, HAH performed the data collection and interpretation of the data. MCRS and HAH verified the coded results. MCRS and HAH analysed the data. MCRS, GOC, HAH contributed to drafting the manuscript. MCRS, GOC, HAH were involved in critical revisions of the manuscript for important intellectual content. All authors approved the final draft of the manuscript.

Funding. This study has no funding.

Competing. The authors declare no conflict of interest.

Ethics statements. Patient consent for publication. Not required.

Data availability statement. The data presented in this study are publicly available at:

SINADEF, MINSA: https://www.datosabiertos.gob.pe/dataset/fallecidos-por-covid-19-ministerio-de-salud-minsa

INEI, Peruvian population: https://www.inei.gob.pe/estadisticas/indice-tematico/population-estimates-and-projections/

Acknowledgments. Not available

References

1. Dhama K, Khan S, Tiwari R, Sircar S, Bhat S, Malik YS, et al. Coronavirus Disease 2019-COVID-19. Clin Microbiol Rev. 2020;33(4):e00028-20.
2. O'Driscoll M, Ribeiro Dos Santos G, Wang L, Cummings DAT, Azman AS, Paireau J, et al. Age-specific mortality and immunity patterns of SARS-CoV-2. Nature. 2021;590(7844):140-145.
3. Bilinski A, Emanuel EJ. COVID-19 and Excess All-Cause Mortality in the US and 18 Comparison Countries. JAMA. 2020;324(20):2100-2102.
4. Chen YH, Glymour MM, Catalano R, Fernandez A, Nguyen T, Kushel M, et al. Excess Mortality in California During the Coronavirus Disease 2019 Pandemic, March to August 2020. JAMA Intern Med. 2021 May 1;181(5):705-707.
5. Banerjee A, Pasea L, Harris S, Gonzalez-Izquierdo A, Torralbo A, Shallcross L, et al. Estimating excess 1-year mortality associated with the COVID-19 pandemic according to underlying conditions and age: a population-based cohort study. Lancet. 2020;395(10238):1715-1725.
6. Kontopantelis E, Mamas MA, Deanfield J, Asaria M, Doran T. Excess mortality in England and Wales during the first wave of the COVID-19 pandemic. J Epidemiol Community Health. 2021;75(3):213-223.
7. Gianicolo EAL, Russo A, Büchler B, Taylor K, Stang A, Blettner M. Gender specific excess mortality in Italy during the COVID-19 pandemic accounting for age. Eur J Epidemiol. 2021 Feb;36(2):213-218.

For peer review only - http://bmjopen.bmj.com/site/about/guidelines.xhtml

8. Palacio-Mejía LS, Wheatley-Fernández JL, Ordóñez-Hernández I, López-Ridaura R, López Gatell-Ramírez H, Hernández-Ávila M, et al. All-cause excess mortality during the Covid-19 pandemic in Mexico. Salud Publica Mex. 2021;63:211-224
9. Locatelli I, Rousson V. A first analysis of excess mortality in Switzerland in 2020. PLoS ONE, 2021;16(6): e0253505
10. Liu J, Zhang L, Yan Y, Zhou Y, Yin P, Qi J, et al. Excess mortality in Wuhan city and other parts of China during the three months of the covid-19 outbreak: findings from nationwide mortality registries. BMJ. 2021;372:n415.
11. Islam N, Shkolnikov VM, Acosta RJ, Klimkin I, Kawachi I, Irizarry RA, et al. Excess deaths associated with covid-19 pandemic in 2020: age and sex disaggregated time series analysis in 29 high income countries. BMJ. 2021;373:n1137.
12. Peruvian Ministry of Health (MINSA). Sala Situaional. COVID-19 Peru. Lima: MINSA; 2021 (accessed on 27 July 2021). Available online: https://covid19.minsa.gob.pe/
13. Peruvian Ministry of Health (MINSA). Excess Mortality. Lima: MINSA; Julay 25, 2021. Available in: https://www.minsa.gob.pe/reunis/data/exceso_mortalidad.asp
14. Ramírez-Soto MC, Arroyo-Hernández H, Ortega-Cáceres G (2021) Sex differences in the incidence, mortality, and fatality of COVID-19 in Peru. PLoS ONE 16(6): e0253193.
15. Munayco C, Chowell G, Tariq A, Undurraga EA, Mizumoto K. Risk of death by age and gender from CoVID-19 in Peru, March-May, 2020. Aging (Albany NY). 2020;12(14):13869–81.
16. Peruvian Ministry of Health (MINSA). COVID-19 deaths. National System of Deaths (SINADEF). Lima: MINSA; 2021 (accessed on 03 June 2021). Available online: https://www.datosabiertos.gob.pe/dataset/fallecidos-por-covid-19-ministerio-de-salud-minsa
17. Karlinsky A, Kobak D. Tracking excess mortality across countries during the COVID-19 pandemic with the World Mortality Dataset. Elife. 2021;10:e69336. doi: 10.7554/eLife.69336.
18. Vestergaard LS, Mølbak K. Timely monitoring of total mortality associated with COVID-19: informing public health and the public. Euro Surveill. 2020;25(34):2001591. https://doi.org/10.2807/1560-7917.ES.2020.25.34.2001591.
19. Woolf SH, Chapman DA, Sabo RT, Weinberger DM, Hill L, Taylor DDH. Excess Deaths From COVID-19 and Other Causes, March-July 2020. *JAMA* 2020;324:1562-4. Doi:10.1001/jama.2020.19545
20. Kontis V, Bennett JE, Rashid T, Parks RM, Pearson-Stuttard J, Guillot M, *et al.* Magnitude, demographics and dynamics of the effect of the first wave of the Covid-19 pandemic on all-cause mortality in 21 industrialized countries. Nat Med. 2020;26:1919-28. https://doi.org/10.10
21. Silva GAE, Jardim BC, Santos C. Excess mortality in Brazil in times of Covid-19. Cien Saude Colet. 2020;25(9):3345-54. https://doi. org/10.1590/1413-81232020259.23642020
22. Gebhard C, Regitz-Zagrosek V, Neuhauser HK, Morgan R, Klein SL. Impact of sex and gender on COVID-19 outcomes in Europe. Biol Sex Differ. 2020; 11(1):29. 11:29. pmid:32450906
23. Abate BB, Kassie AM, Kassaw MW, Aragie TG, Masresha SA. Sex difference in coronavirus disease (COVID-19): a systematic review and meta-analysis. BMJ Open. 2020 Oct 6;10(10):e040129. pmid:33028563
24. Islam N, Shkolnikov VM, Acosta RJ, Klimkin I, Kawachi I, Irizarry RA. Excess deaths associated with covid-19 pandemic in 2020: age and sex disaggregated time series analysis in 29 high income countriesBMJ 2021;373:n1137. http://dx.doi.org/10.1136/bmj.n1137

For peer review only - http://bmjopen.bmj.com/site/about/guidelines.xhtml

25. Velásquez Aníbal. La carga de enfermedad y lesiones en el Perú y las prioridades del plan esencial de aseguramiento universal. Rev Perú Med Exp Salud Publica. 2009; 26(2): 222-231.

26. Yamada G, Castillo-Salgado C, Jones-Smith JC, Moulton LH. Differences in magnitude and rate of change in adult obesity distribution by age and sex in Mexico, Colombia and Peru, 2005-2010. Public Health Nutr. 2019 Mar;22(4):757-763. doi: 10.1017/S1368980018002343.

27. Zhou F, Yu T, Du R, et al. Clinical course and risk factors for mortality of adult inpatients with COVID-19 in Wuhan, China: a retrospective cohort study. Lancet. 2020;395(10229):1054–1062. pmid:32171076

28. Seclén SN, Nunez-Robles E, Yovera-Aldana M, Arias-Chumpitaz A. Incidence of COVID-19 infection and prevalence of diabetes, obesity and hypertension according to altitude in Peruvian population. Diabetes Res Clin Pract. 2020 Nov;169:108463. doi: 10.1016/j.diabres.2020.108463.

29. Álvarez-Antonio C, Meza-Sánchez G, Calampa C, Casanova W, Carey C, Alava F. et al. Seroprevalence of anti-SARS-CoV-2 antibodies in Iquitos, Peru in July and August, 2020: a population-based study. Lancet Glob Health. 2021 Jul;9(7):e925-e931. doi: 10.1016/S2214-109X(21)00173-X.

30. Reyes-Vega MF, Soto-Cabezas MG, Cárdenas F, Martel KS, Valle A, Valverde J. et al. SARS-CoV-2 prevalence associated to low socioeconomic status and overcrowding in an LMIC megacity: A population-based seroepidemiological survey in Lima, Peru. EClinicalMedicine. 2021;34:100801. doi: 10.1016/j.eclinm.2021.100801.

Legends:

Figure 1. Excess numbers of deaths (A) and excess death rates (B) in men and women in Peru stratified by age.

Figure 2. ORs for excess death rates stratified by age in men (A) and women (B) in Peru.

Figure 3. Observed, expected, and excess death (A) and excess death rates (B) stratified by region in men and women in Peru.

Figure 4. ORs for excess death rates according to region in men (A) and women (B) in Peru.

Figure 5. Temporal distribution of excess deaths (%) in men and women in Peru.

Figure 6. Temporal distribution of excess deaths according to region in men (A) and women (B) in Peru.

For peer review only - http://bmjopen.bmj.com/site/about/guidelines.xhtml

STROBE Statement—checklist of items that should be included in reports of observational studies

	Item No	Recommendation	Page No
**Title and abstract**	1	(*a*) Indicate the study's design with a commonly used term in the title or the abstract	1
		(*b*) Provide in the abstract an informative and balanced summary of what was done and what was found	2
**Introduction**			
Background/rationale	2	Explain the scientific background and rationale for the investigation being reported	4
Objectives	3	State specific objectives, including any prespecified hypotheses	4
**Methods**			
Study design	4	Present key elements of study design early in the paper	4
Setting	5	Describe the setting, locations, and relevant dates, including periods of recruitment, exposure, follow-up, and data collection	5
Participants	6	(*a*) *Cohort study*—Give the eligibility criteria, and the sources and methods of selection of participants. Describe methods of follow-up<br>*Case-control study*—Give the eligibility criteria, and the sources and methods of case ascertainment and control selection. Give the rationale for the choice of cases and controls<br>*Cross-sectional study*—Give the eligibility criteria, and the sources and methods of selection of participants	NA
		(*b*) *Cohort study*—For matched studies, give matching criteria and number of exposed and unexposed<br>*Case-control study*—For matched studies, give matching criteria and the number of controls per case	NA
Variables	7	Clearly define all outcomes, exposures, predictors, potential confounders, and effect modifiers. Give diagnostic criteria, if applicable	5
Data sources/ measurement	8*	For each variable of interest, give sources of data and details of methods of assessment (measurement). Describe comparability of assessment methods if there is more than one group	*5*
Bias	9	Describe any efforts to address potential sources of bias	5
Study size	10	Explain how the study size was arrived at	5

For peer review only - http://bmjopen.bmj.com/site/about/guidelines.xhtml

For peer review only

Quantitative variables	11	Explain how quantitative variables were handled in the analyses. If applicable, describe which groupings were chosen and why	5
Statistical methods	12	(*a*) Describe all statistical methods, including those used to control for confounding	5
		(*b*) Describe any methods used to examine subgroups and interactions	5
		(*c*) Explain how missing data were addressed	5
		(*d*) *Cohort study*—If applicable, explain how loss to follow-up was addressed<br>*Case-control study*—If applicable, explain how matching of cases and controls was addressed<br>*Cross-sectional study*—If applicable, describe analytical methods taking account of sampling strategy	NA
		(*e*) Describe any sensitivity analyses	NA

Continued on next page

For peer review only

**Results**			
Participants	13*	(a) Report numbers of individuals at each stage of study—eg numbers potentially eligible, examined for eligibility, confirmed eligible, included in the study, completing follow-up, and analysed	5
		(b) Give reasons for non-participation at each stage	5
		(c) Consider use of a flow diagram	5
Descriptive data	14*	(a) Give characteristics of study participants (eg demographic, clinical, social) and information on exposures and potential confounders	5
		(b) Indicate number of participants with missing data for each variable of interest	5
		(c) *Cohort study*—Summarise follow-up time (eg, average and total amount)	NA
Outcome data	15*	*Cohort study*—Report numbers of outcome events or summary measures over time	*NA*
		Case-control study—Report numbers in each exposure category, or summary measures of exposure	*NA*
		Cross-sectional study—Report numbers of outcome events or summary measures	*NA*
Main results	16	(*a*) Give unadjusted estimates and, if applicable, confounder-adjusted estimates and their precision (eg, 95% confidence interval). Make clear which confounders were adjusted for and why they were included	6
		(*b*) Report category boundaries when continuous variables were categorized	6
		(*c*) If relevant, consider translating estimates of relative risk into absolute risk for a meaningful time period	6
Other analyses	17	Report other analyses done—eg analyses of subgroups and interactions, and sensitivity analyses	6
**Discussion**			
Key results	18	Summarise key results with reference to study objectives	6
Limitations	19	Discuss limitations of the study, taking into account sources of potential bias or imprecision. Discuss both direction and magnitude of any potential bias	7
Interpretation	20	Give a cautious overall interpretation of results considering objectives, limitations, multiplicity of analyses, results from similar studies, and other relevant evidence	7
Generalisability	21	Discuss the generalisability (external validity) of the study results	7

Other information

For peer review only - http://bmjopen.bmj.com/site/about/guidelines.xhtml

Funding	22	Give the source of funding and the role of the funders for the present study and, if applicable, for the original study on which the present article is based	8

*Give information separately for cases and controls in case-control studies and, if applicable, for exposed and unexposed groups in cohort and cross-sectional studies.

Note: An Explanation and Elaboration article discusses each checklist item and gives methodological background and published examples of transparent reporting. The STROBE checklist is best used in conjunction with this article (freely available on the Web sites of PLoS Medicine at http://www.plosmedicine.org/, Annals of Internal Medicine at http://www.annals.org/, and Epidemiology at http://www.epidem.com/). Information on the STROBE Initiative is available at www.strobe-statement.org.
NA: Not available

For peer review only

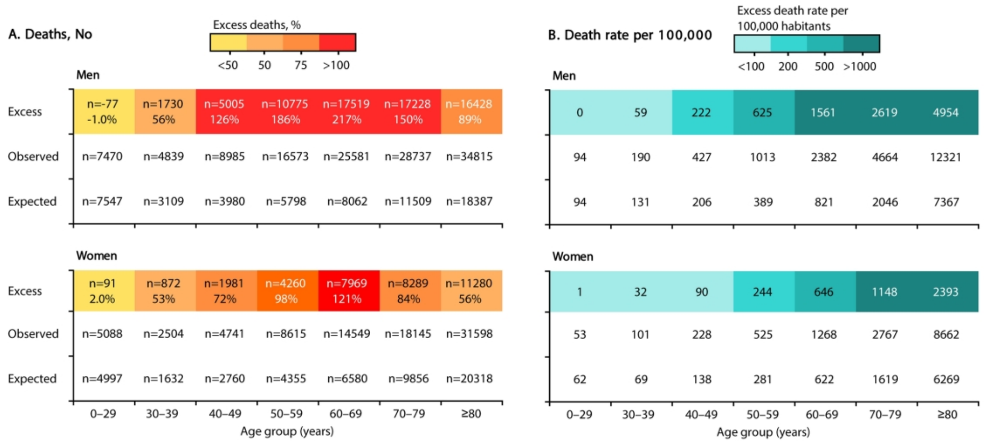

Figure 1. Excess numbers of deaths (A) and excess death rates (B) in men and women in Peru stratified by age.

190x87mm (300 x 300 DPI)

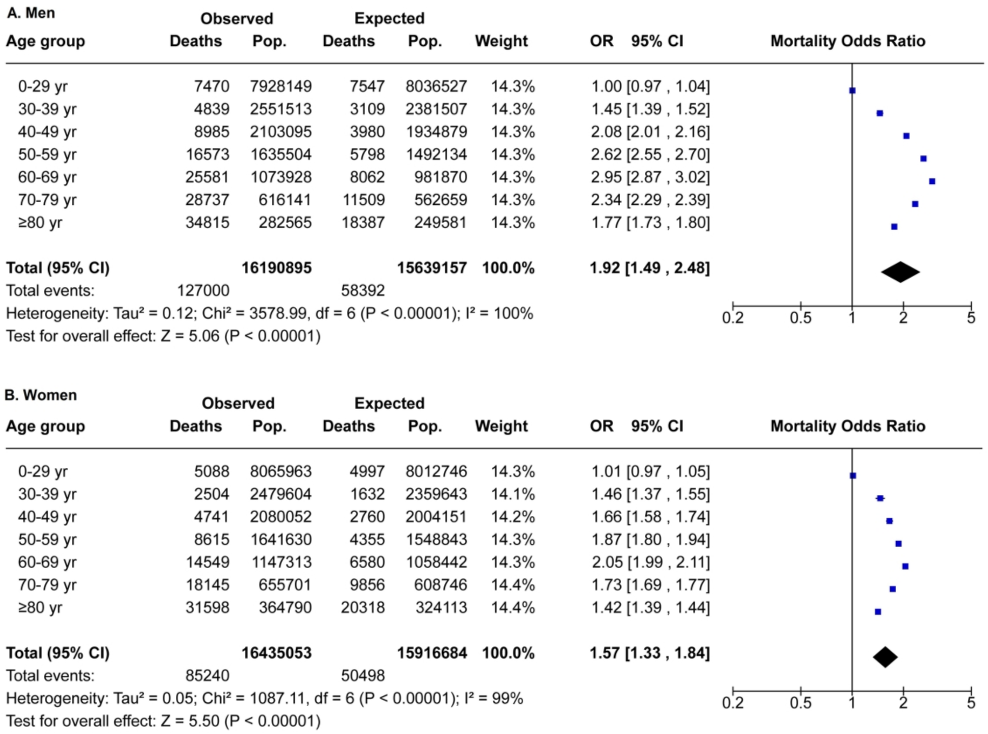

Figure 2. ORs for excess death rates stratified by age in men (A) and women (B) in Peru.

190x145mm (300 x 300 DPI)

A. Deaths

Region	Men Expected	Men Observed	Men Excess	Women Expected	Women Observed	Women Excess
Lima	17273	51668	34395 (199%)	14986	33683	18697 (125%)
Callao	2453	5951	3498 (143%)	1936	3752	1816 (94%)
Pasco	340	675	335 (98%)	282	539	257 (91%)
Tumbes	511	1073	562 (110%)	384	704	320 (83%)
Piura	2921	6921	4000 (137%)	2463	4393	1930 (78%)
Loreto	1250	2769	1519 (122%)	947	1678	731 (77%)
Arequipa	3088	6897	3809 (123%)	2665	4633	1968 (74%)
Ucayali	997	1852	855 (86%)	726	1196	470 (65%)
Madre de Dios	379	729	350 (93%)	213	349	136 (64%)
Ica	2226	4651	2425 (109%)	1992	3114	1122 (56%)
Moquegua	427	977	550 (129%)	354	552	198 (56%)
San Martin	1495	2591	1096 (73%)	1124	1621	497 (44%)
Apurimac	805	1151	346 (43%)	724	1041	317 (44%)
La Libertad	4103	7433	3330 (81%)	3664	5181	1517 (41%)
Junin	2968	4888	1920 (65%)	2566	3617	1051 (41%)
Ancash	2786	5058	2272 (82%)	2517	3519	1002 (40%)
Cajamarca	1655	2943	1288 (78%)	1576	2152	576 (37%)
Huanuco	1545	2240	695 (45%)	1412	1798	386 (27%)
Ayacucho	903	1409	506 (56%)	882	1116	234 (27%)
Huancavelica	937	1297	360 (38%)	928	1129	201 (22%)
Cusco	3212	4317	1105 (34%)	2747	3341	594 (22%)
Puno	2761	3988	1227 (44%)	2521	3028	507 (20%)
Amazonas	477	681	204 (43%)	396	438	42 (11%)
Tacna	719	1086	367 (51%)	626	668	42 (7%)
Lambayeque	1865	3439	1574 (84%)	1691	1801	110 (7%)

Excess deaths, %: <50; 51-75; 76-99; ≥100

B. Mortality

Region	Men Expected	Men Observed	Men Excess	Women Expected	Women Observed	Women Excess
Lima	353	1009	656	284	611	328
Callao	468	1082	614	349	647	298
Moquegua	431	950	519	405	614	209
Arequipa	441	937	496	366	608	242
Ica	482	951	469	432	640	208
Tumbes	393	791	398	346	608	262
Piura	295	671	376	250	432	182
Ancash	480	850	370	438	601	163
Madre de Dios	414	742	328	305	462	157
La Libertad	427	743	316	375	510	135
Loreto	242	521	280	196	338	142
Junin	447	720	274	383	530	147
Ucayali	342	602	260	272	425	153
Pasco	243	481	238	214	409	195
Lambayeque	302	539	237	259	268	9
San Martin	326	546	220	275	381	106
Huancavelica	496	704	209	496	623	127
Puno	452	652	200	403	483	81
Huanuco	404	583	179	377	478	101
Tacna	402	577	175	356	365	9
Cajamarca	230	405	175	219	296	77
Apurimac	369	522	153	347	495	148
Cusco	481	629	147	420	498	78
Ayacucho	269	412	143	273	342	69
Amazonas	221	310	89	195	212	17

Mortality Rate per 100,000 hab.: <100; 100-199; 200-299; ≥300

Figure 3. Observed, expected, and excess death (A) and excess death rates (B) stratified by region in men and women in Peru.

183x213mm (300 x 300 DPI)

A. Men

Department	Observed Deaths	Observed Pop.	Expected Deaths	Expected Pop.	Weight	Odds ratio M-H, Random, 95% CI
Pasco	675	140252	340	139884	3.9%	1.98 [1.74 , 2.26]
Madre de Dios	729	98215	379	91405	3.9%	1.80 [1.59 , 2.03]
Amazonas	681	219801	477	216208	3.9%	1.41 [1.25 , 1.58]
Moquegua	977	102855	427	99015	3.9%	2.21 [1.98 , 2.48]
Tumbes	1073	135675	511	130076	3.9%	2.02 [1.82 , 2.25]
Tacna	1086	188152	719	178933	4.0%	1.44 [1.31 , 1.58]
Apurimac	1151	220370	805	218355	4.0%	1.42 [1.30 , 1.55]
Huancavelica	1297	184121	937	189070	4.0%	1.42 [1.31 , 1.55]
Ayacucho	1409	341951	903	336027	4.0%	1.54 [1.41 , 1.67]
Ucayali	1852	307596	997	291731	4.0%	1.77 [1.64 , 1.91]
Loreto	2769	531000	1250	517031	4.0%	2.16 [2.02 , 2.31]
Huanuco	2240	384345	1545	382763	4.0%	1.45 [1.36 , 1.54]
San Martin	2591	474458	1495	458180	4.0%	1.68 [1.57 , 1.79]
Cajamarca	2943	727265	1655	719183	4.0%	1.76 [1.66 , 1.87]
Lambayeque	3439	638228	1865	617737	4.0%	1.79 [1.69 , 1.89]
Ica	4651	488836	2226	461834	4.0%	1.98 [1.89 , 2.09]
Puno	3988	611616	2761	610794	4.0%	1.45 [1.38 , 1.52]
Callao	5951	550046	2453	524243	4.0%	2.33 [2.22 , 2.44]
Ancash	5058	594832	2786	580457	4.0%	1.78 [1.70 , 1.86]
Cusco	4317	686543	3212	667014	4.0%	1.31 [1.25 , 1.37]
Junin	4888	678494	2968	664177	4.0%	1.62 [1.54 , 1.69]
Piura	6921	1030975	2921	989817	4.0%	2.28 [2.19 , 2.38]
Arequipa	6897	735707	3088	700017	4.0%	2.14 [2.05 , 2.23]
La Libertad	7433	1000002	4103	960186	4.1%	1.75 [1.68 , 1.81]
Lima	51668	5119560	17273	4895018	4.1%	2.88 [2.83 , 2.93]
**Total (95% CI)**		**16190895**		**15639155**	**100.0%**	**1.78 [1.59 , 1.99]**
Total events:	126684		58096			

Heterogeneity: Tau² = 0.08; Chi² = 2807.31, df = 24 (P < 0.00001); I² = 99%
Test for overall effect: Z = 9.94 (P < 0.00001)

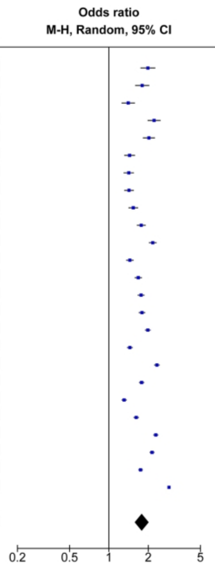

B. Women

Department	Observed Deaths	Observed Pop.	Expected Deaths	Expected Pop.	Weight	Odds ratio M-H, Random, 95% CI
Madre de Dios	349	75596	213	69936	3.7%	1.52 [1.28 , 1.80]
Pasco	539	131652	282	131748	3.8%	1.92 [1.66 , 2.21]
Amazonas	438	207005	396	203514	3.8%	1.09 [0.95 , 1.25]
Moquegua	552	89885	354	87315	3.8%	1.52 [1.33 , 1.74]
Tumbes	704	115846	384	111202	3.9%	1.76 [1.56 , 2.00]
Tacna	668	182822	626	175653	3.9%	1.03 [0.92 , 1.14]
Apurimac	1041	210366	724	208854	4.0%	1.43 [1.30 , 1.57]
Ucayali	1196	281514	726	266976	4.0%	1.56 [1.43 , 1.72]
Ayacucho	1116	326262	882	322858	4.0%	1.25 [1.15 , 1.37]
Huancavelica	1129	181196	928	187225	4.0%	1.26 [1.15 , 1.37]
Loreto	1678	496559	947	483076	4.0%	1.73 [1.59 , 1.87]
San Martin	1621	425190	1124	408504	4.0%	1.39 [1.29 , 1.50]
Huanuco	1798	375922	1412	374502	4.0%	1.27 [1.18 , 1.36]
Lambayeque	1801	672557	1691	652268	4.1%	1.03 [0.97 , 1.10]
Cajamarca	2152	726446	1576	718755	4.1%	1.35 [1.27 , 1.44]
Ica	3114	486346	1992	461257	4.1%	1.49 [1.40 , 1.57]
Callao	3752	579808	1936	554381	4.1%	1.86 [1.76 , 1.96]
Puno	3028	626381	2521	625717	4.1%	1.20 [1.14 , 1.27]
Ancash	3519	585806	2517	574696	4.1%	1.37 [1.31 , 1.45]
Junin	3617	682973	2566	670614	4.1%	1.39 [1.32 , 1.46]
Cusco	3341	670532	2747	653196	4.1%	1.19 [1.13 , 1.25]
Piura	4393	1016979	2463	984142	4.1%	1.73 [1.65 , 1.82]
Arequipa	4633	761731	2665	728480	4.1%	1.67 [1.59 , 1.75]
La Libertad	5181	1016769	3664	977944	4.1%	1.36 [1.31 , 1.42]
Lima	33683	5508910	14986	5283870	4.1%	2.16 [2.12 , 2.21]
**Total (95% CI)**		**16435053**		**15916683**	**100.0%**	**1.43 [1.30 , 1.59]**
Total events:	85043		50322			

Heterogeneity: Tau² = 0.07; Chi² = 1765.90, df = 24 (P < 0.00001); I² = 99%
Test for overall effect: Z = 6.97 (P < 0.00001)

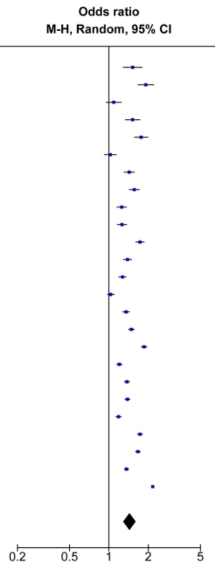

Figure 4. ORs for excess death rates according to region in men (A) and women (B) in Peru.

162x222mm (300 x 300 DPI)

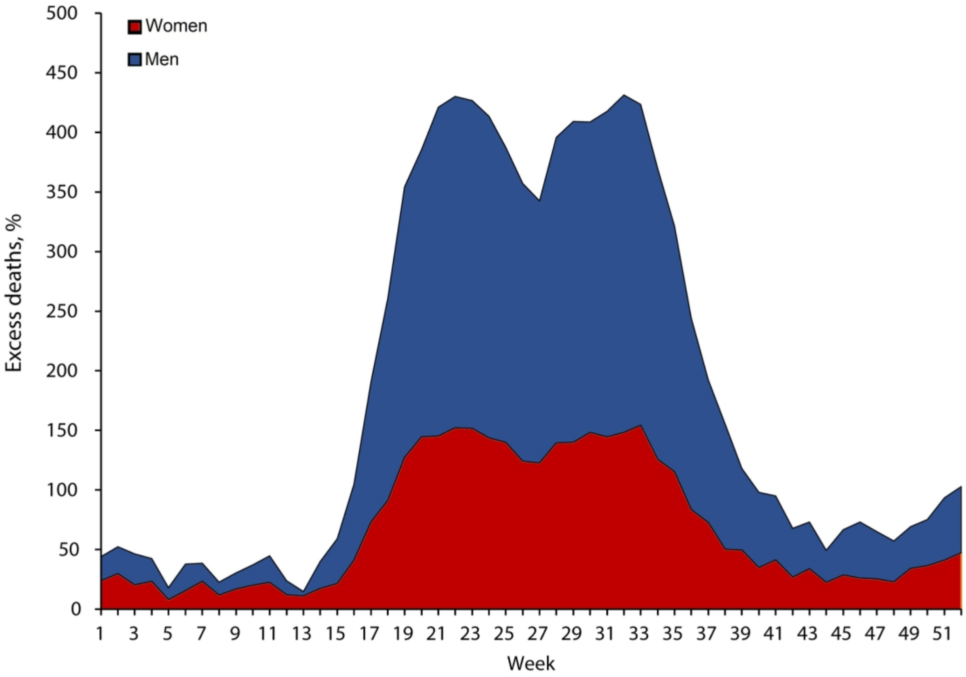

Figure 5. Temporal distribution of excess deaths (%) in men and women in Peru.

154x110mm (300 x 300 DPI)

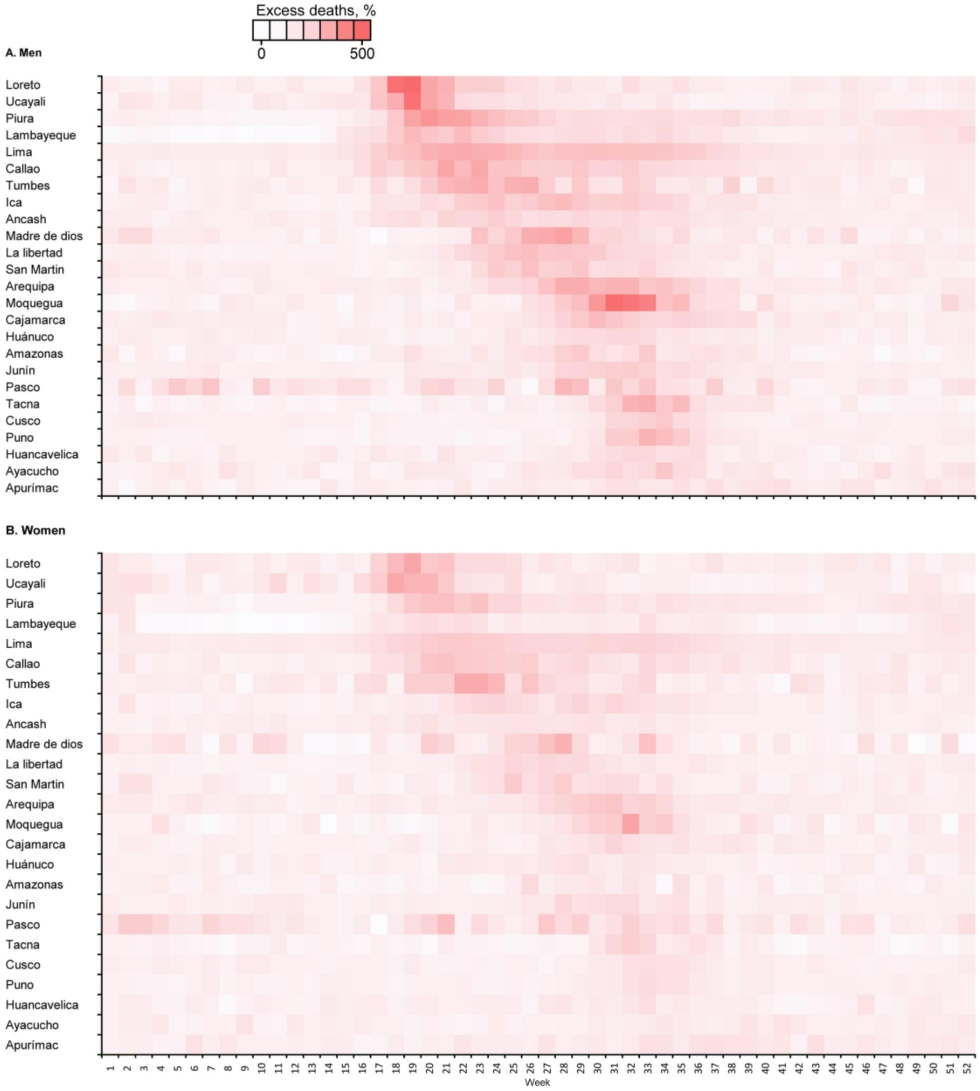

Figure 6. Temporal distribution of excess deaths according to region in men (A) and women (B) in Peru.

181x198mm (300 x 300 DPI)

STROBE Statement—checklist of items that should be included in reports of observational studies

	Item No	Recommendation	Page No
**Title and abstract**	1	(*a*) Indicate the study's design with a commonly used term in the title or the abstract	1
		(*b*) Provide in the abstract an informative and balanced summary of what was done and what was found	2
**Introduction**			
Background/rationale	2	Explain the scientific background and rationale for the investigation being reported	4
Objectives	3	State specific objectives, including any prespecified hypotheses	4
**Methods**			
Study design	4	Present key elements of study design early in the paper	4
Setting	5	Describe the setting, locations, and relevant dates, including periods of recruitment, exposure, follow-up, and data collection	5
Participants	6	(*a*) *Cohort study*—Give the eligibility criteria, and the sources and methods of selection of participants. Describe methods of follow-up<br>*Case-control study*—Give the eligibility criteria, and the sources and methods of case ascertainment and control selection. Give the rationale for the choice of cases and controls<br>*Cross-sectional study*—Give the eligibility criteria, and the sources and methods of selection of participants	NA
		(*b*) *Cohort study*—For matched studies, give matching criteria and number of exposed and unexposed<br>*Case-control study*—For matched studies, give matching criteria and the number of controls per case	NA
Variables	7	Clearly define all outcomes, exposures, predictors, potential confounders, and effect modifiers. Give diagnostic criteria, if applicable	5
Data sources/ measurement	8*	For each variable of interest, give sources of data and details of methods of assessment (measurement). Describe comparability of assessment methods if there is more than one group	*5*
Bias	9	Describe any efforts to address potential sources of bias	5
Study size	10	Explain how the study size was arrived at	5

For peer review only

Quantitative variables	11	Explain how quantitative variables were handled in the analyses. If applicable, describe which groupings were chosen and why	5
Statistical methods	12	(*a*) Describe all statistical methods, including those used to control for confounding	5
		(*b*) Describe any methods used to examine subgroups and interactions	5
		(*c*) Explain how missing data were addressed	5
		(*d*) *Cohort study*—If applicable, explain how loss to follow-up was addressed<br>*Case-control study*—If applicable, explain how matching of cases and controls was addressed<br>*Cross-sectional study*—If applicable, describe analytical methods taking account of sampling strategy	NA
		(*e*) Describe any sensitivity analyses	NA

Continued on next page

For peer review only

Results

Participants	13*	(a) Report numbers of individuals at each stage of study—eg numbers potentially eligible, examined for eligibility, confirmed eligible, included in the study, completing follow-up, and analysed	5
		(b) Give reasons for non-participation at each stage	5
		(c) Consider use of a flow diagram	5
Descriptive data	14*	(a) Give characteristics of study participants (eg demographic, clinical, social) and information on exposures and potential confounders	5
		(b) Indicate number of participants with missing data for each variable of interest	5
		(c) *Cohort study*—Summarise follow-up time (eg, average and total amount)	NA
Outcome data	15*	*Cohort study*—Report numbers of outcome events or summary measures over time	*NA*
		Case-control study—Report numbers in each exposure category, or summary measures of exposure	*NA*
		Cross-sectional study—Report numbers of outcome events or summary measures	*NA*
Main results	16	(*a*) Give unadjusted estimates and, if applicable, confounder-adjusted estimates and their precision (eg, 95% confidence interval). Make clear which confounders were adjusted for and why they were included	6
		(*b*) Report category boundaries when continuous variables were categorized	6
		(*c*) If relevant, consider translating estimates of relative risk into absolute risk for a meaningful time period	6
Other analyses	17	Report other analyses done—eg analyses of subgroups and interactions, and sensitivity analyses	6

Discussion

Key results	18	Summarise key results with reference to study objectives	6
Limitations	19	Discuss limitations of the study, taking into account sources of potential bias or imprecision. Discuss both direction and magnitude of any potential bias	7
Interpretation	20	Give a cautious overall interpretation of results considering objectives, limitations, multiplicity of analyses, results from similar studies, and other relevant evidence	7
Generalisability	21	Discuss the generalisability (external validity) of the study results	7

Other information

For peer review only

Funding	22	Give the source of funding and the role of the funders for the present study and, if applicable, for the original study on which the present article is based	8

*Give information separately for cases and controls in case-control studies and, if applicable, for exposed and unexposed groups in cohort and cross-sectional studies.

Note: An Explanation and Elaboration article discusses each checklist item and gives methodological background and published examples of transparent reporting. The STROBE checklist is best used in conjunction with this article (freely available on the Web sites of PLoS Medicine at http://www.plosmedicine.org/, Annals of Internal Medicine at http://www.annals.org/, and Epidemiology at http://www.epidem.com/). Information on the STROBE Initiative is available at www.strobe-statement.org.

NA: Not available

For peer review only

BMJ Open

Excess all-cause deaths stratified by sex and age in Peru: a time series analysis during the COVID-19 pandemic

Journal:	*BMJ Open*
Manuscript ID	bmjopen-2021-057056.R1
Article Type:	Original research
Date Submitted by the Author:	13-Dec-2021
Complete List of Authors:	Ramírez-Soto, Max Carlos; Universidad Peruana Cayetano Heredia; Universidad Tecnológica del Perú<br>Ortega-Cáceres, Gutia; Universidad Ricardo Palma<br>Arroyo-Hernández, Hugo; Instituto Nacional de Salud
**Primary Subject Heading**:	Epidemiology
Secondary Subject Heading:	Epidemiology, Health policy
Keywords:	COVID-19, EPIDEMIOLOGY, PUBLIC HEALTH

SCHOLARONE™
Manuscripts

I, the Submitting Author has the right to grant and does grant on behalf of all authors of the Work (as defined in the below author licence), an exclusive licence and/or a non-exclusive licence for contributions from authors who are: i) UK Crown employees; ii) where BMJ has agreed a CC-BY licence shall apply, and/or iii) in accordance with the terms applicable for US Federal Government officers or employees acting as part of their official duties; on a worldwide, perpetual, irrevocable, royalty-free basis to BMJ Publishing Group Ltd ("BMJ") its licensees and where the relevant Journal is co-owned by BMJ to the co-owners of the Journal, to publish the Work in this journal and any other BMJ products and to exploit all rights, as set out in our licence.

The Submitting Author accepts and understands that any supply made under these terms is made by BMJ to the Submitting Author unless you are acting as an employee on behalf of your employer or a postgraduate student of an affiliated institution which is paying any applicable article publishing charge ("APC") for Open Access articles. Where the Submitting Author wishes to make the Work available on an Open Access basis (and intends to pay the relevant APC), the terms of reuse of such Open Access shall be governed by a Creative Commons licence – details of these licences and which Creative Commons licence will apply to this Work are set out in our licence referred to above.

Other than as permitted in any relevant BMJ Author's Self Archiving Policies, I confirm this Work has not been accepted for publication elsewhere, is not being considered for publication elsewhere and does not duplicate material already published. I confirm all authors consent to publication of this Work and authorise the granting of this licence.

For peer review only

Excess all-cause deaths stratified by sex and age in Peru: a time series analysis during the COVID-19 pandemic

Max Carlos Ramírez-Soto [1,2,*], Gutia Ortega-Cáceres [3], Hugo Arroyo-Hernández [4]

[1] School of Public Health and Administration, Universidad Peruana Cayetano Heredia, Lima, Peru

[2] Facultad de Ciencias de la Salud, Universidad Tecnológica del Perú, Lima, Peru

[3] Escuela de Posgrado, Universidad Ricardo Palma, Lima, Peru

[4] Oficina General de Información y Sistemas, Instituto Nacional de Salud, Lima, Peru

***Correspondence:**

Max Carlos Ramírez-Soto;

School of Public Health and Administration, Universidad Peruana Cayetano Heredia, Lima, Peru

maxcrs22@gmail.com; Tel.: +51 984887238

Conflicts of Interest: The authors declare no conflict of interest.

Funding: Please add: “This research received no external funding”

Words: 3101

Figures: 07

For peer review only - http://bmjopen.bmj.com/site/about/guidelines.xhtml

For peer review only

Abstract

Background. In this study, we estimated the excess all-causes deaths and excess death rates during the COVID-19 pandemic in 25 Peruvian regions, stratified by sex and age groups.

Design. Time series analysis.

Setting. 25 Peruvian regions with complete mortality data.

Participants. Annual all cause official mortality data set from the Sistema Informatico Nacional de Defunciones (SINADEF) at the Ministry of Health of Peru for 2017-20, disaggregated by age and sex.

Main outcome measures. Excess deaths and the excess death rates (observed deaths vs. expected deaths) in 2020, by sex and age (0-29, 30-39, 40-49, 50-59, 60-69, 70-79 and ≥80 years), estimated using P-score. The odds ratios (OR) for excess mortality were summarized with a random effects meta-analysis.

Results. In the period between January and December 2020, we estimated an excess of 68,608 (117%) deaths in men and 34,742 (69%) deaths in women, corresponding to an excess death rate of 424 per 100,000 men and 211 per 100,000 women compared to the expected mortality rates. The number of excess deaths increased with age and was higher in men aged 60–69 years (217%) compared with women (121%). Men between the ages of 40 and 79 years experienced twice the rate of excess deaths compared with the rate expected. In eight regions, the excess deaths were higher than 100% in men, and in seven regions the excess deaths were higher than 70% in women. Men in eight regions and women in one region had two times increased odds of excess death than the expected mortality. There were differences in excess mortality according to the temporal distribution by epidemiological week.

Conclusion. Approximately one hundred thousand excess all-cause deaths occurred in 2020 in Peru. The age-stratified excess death rates were higher in men than in women. There were strong excesses in geographical and temporal mortality patterns according to region.

Keywords: excess mortality; sex; mortality rate; COVID-19; Peru

For peer review only - http://bmjopen.bmj.com/site/about/guidelines.xhtml

Strengths and limitations of this study

- The main strength of our study was the large number of deaths included (302,177 in men and 236,733 in women) for estimating the excess all-cause deaths.
- Although our statistical analysis was based in P-score, our estimates of excess all-cause deaths are similar to those previously developed using more sophisticated statistical analyses.
- The simplicity in the analysis of excess mortality allows improving the opportunity to use the findings in epidemiological surveillance and their interpretation by those responsible for the formulation of public policies and health authorities at different levels.
- Although SINADEF is an official database, there may have been delays in its registration due to a lack of notification of non-hospital or manually registered deaths.
- We present the results of a descriptive analysis and so we are not able to comment on causality in excess deaths.

For peer review only

Introduction

Following the outbreak of COVID-19 in China in 2019, SARS-CoV-2 spread rapidly worldwide.[1] During the following weeks, there was a rapidly increase in the incidence and mortality rates of COVID-19 in European countries, the USA, and Latin American countries.[2] Previous studies on excess deaths (the gap between observed and expected deaths) during the COVID-19 pandemic have found that COVID-19-related deaths and deaths from other diseases increased along with age and were higher in men 60 years of age and older compared with women.[3-10] More recently, the assessment of the direct and indirect effects of the COVID-19 pandemic on mortality has revealed that approximately one million excess deaths occurred in 2020 in 29 high-income countries. Additionally, these excess death rates were higher in men than in women in almost all countries.[11]

Peru is one of the countries that has been worst affected by the COVID-19 pandemic. The first cases in Peru were reported in March 2020. During the following weeks, rapidly increasing numbers of cases and fatalities were observed across many Peru regions. By the end of December 2020, about 1.0 million confirmed COVID-19 cases and more than 90,000 deaths had been officially reported.[12] In Peru, data on excess all-cause deaths have been reported, but analyses have not accounted for age and sex standardization. From 29 March 2020 (week 14), a significant rate of excess deaths in comparison with the expected deaths based on the figures from 2017 to 2019 has been observed at the national level, with this rate reaching the highest level in week 32 and then decreasing.[13] This increase in all-cause deaths is concomitant with the COVID-19 pandemic, as it has previously been shown that COVID-19 mortality rates increase with age and are high in men aged 60 years and over.[14,15] Despite these findings, to date, the excess mortality stratified by sex, age, and region is not known at the national level. The assessment of the impact of the COVID-19 pandemic on mortality in Peru should include the analysis of both the direct effect of the pandemic on deaths caused by COVID-19 and the indirect effect of the pandemic on deaths unrelated to COVID-19, since patients with chronic conditions may have been turned away from the health care system because of concerns relating to COVID-19 infection and social and economic changes.

In this study, we estimate the excess all-cause deaths and the excess death rate during the first year of the COVID-19 pandemic in 25 Peruvian regions, stratified by sex, age, group, and epidemiological week.

Methods

Study design

We performed an ecological study following the Strengthening the Reporting of Observational Studies in Epidemiology (STROBE) reporting guidelines.[16] This study is a time series analysis of annual data on all-cause mortality obtained from 25 Peruvian regions official, valid, and complete mortality data between 2017 and 2020 disaggregated by age and sex. The Peruvian regions are first-level administrative divisions of the country.

Data collection

We using weekly death data from the Sistema Informatico Nacional de Defunciones (SINADEF) at the Ministry of Health of Peru.[17] The source data (SINADEF) are in Spanish language. We used deaths registers for 25 Peruvian regions from the 01 January through to 31 December 2020 (1–52 epidemiological weeks) and the preceding 3 years (2017–2019). We converted daily data into ISO weeks. SINADEF is a computer application. It registers data on deaths, generates death certificates, and creates a statistical report; it includes fetal deaths and

For peer review only - http://bmjopen.bmj.com/site/about/guidelines.xhtml

the deaths of unidentified persons.[17] Open access data includes information stratified by age/sex, the basic cause of death, the year, the International Classification of Diseases 10th Revision (ICD-10), and the geographic distribution. Other information is not available in SINADEF. All-cause deaths where the underlying cause was known were available, with information on region, age group (0–29, 30–39, 40–49, 50–59, 60–69, 70–79, and 80 and over), and sex included. Records where the place of residence, age, sex, or year was missing were excluded.

Patient and public involvement

We used publicly available death statistics, therefore, there was no direct patient or public involvement.

Ethics statements

This study does not involve human participants. Weekly death data were obtained of SINADEF. In SINADEF, all open access data are fully anonymous and are published as part of routine surveillance (https://www.datosabiertos.gob.pe/dataset/informaci%C3%B3n-de-fallecidos-del-sistema-inform%C3%A1tico-nacional-de-defunciones-sinadef-ministerio). Therefore, this study was exempt from review by an ethics board.

Excess mortality analysis

We estimated the excess deaths and the excess death rates (per 100,000 inhabitants) according to sex and age, as well as in each region by sex. To make comparisons with other countries, we measure excess mortality using P-score [(Observed deaths–Expected deaths)/Expected deaths*100%],[8] i.e., the percentage difference between the observed (weekly or monthly deaths in 2020) and expected number of deaths in 2020 (the average number of deaths in the same period over the years 2017–2019) by sex, age, region and epidemiological week. Mortality rates per 100,000 inhabitants for both sexes were also calculated by dividing the number of deaths per region, standardized by the estimated population of each region. We calculated the expected mortality rate for the year 2020 (the average number of deaths in the same period over the years 2017–2019) and the mortality rate observed in 2020 for both sexes according to age and region (per 100,000 inhabitants). The excess death rate was calculated using the difference between the mortality rate observed in 2020 and the mortality rate expected in 2020 (based on the years 2017–2019). Population counts used for calculating the mortality rates were obtained from projections from Peru's National Institute of Statistics and Informatic (INEI, in Spanish).[18] In addition, odds ratios (ORs) with 95% confidence intervals (CIs) for mortality observed vs. mortality expected according to sex were estimated separately for each age stratum and region. These results were summarized in a random-effects meta-analysis with individual reports weighted using the weights indicated. The Mantel–Haenszel methods were used to calculate the random effects estimates. P<0.05 was considered statistically significant. The meta-analysis was performed in RevMan 5, Cochrane.

Results

From January 1, 2017, to December 31, 2020, a total of 540,802 all-cause deaths occurred in Peru. Of these deaths, we included a total of 302,177 and 236,733 deaths in men and women, respectively (Figure 1).

Excess death and excess death rates by sex and age

Between January 1, 2020, and December 31, 2020, a total of 127,000 all-cause deaths (MR 784 deaths per 100,000 men) were reported in men. In comparison with the expected 58,392 deaths (MR 361 per 100,000 men), the number of excess deaths during the same period was 68,608 (117%), and the excess death rate was 424 deaths per 100,000 men. During the

For peer review only - http://bmjopen.bmj.com/site/about/guidelines.xhtml

same period, a total of 85,240 (MR, 519 deaths per 100,000 women) all-cause deaths were reported in women. In comparison with the expected 50,498 deaths (MR, 307 deaths per 100,000 women), the number of excess deaths during the same period was 34,742 (69%), and the excess death rate was 211 deaths per 100,000 women.

The number of excess deaths increased with age and was higher in men aged 60–69 years (217%) compared with women (121%) (Figure 2A). The all-cause mortality rate in relation to the expected death rate increased with age and was higher in individuals aged 80 years or more in both sexes (Figure 2B). In addition, the increase in the all-cause mortality rate was more than twice as high in men compared with that in women.

ORs for excess death rates stratified by sex and age

In Figure 3, our meta-analysis shows a continuous age-dependent increase in the number of excess deaths in men and women. Overall, men and women had 2.08 (95% CI, 1.59–2.73) and 1.67 (95% CI, 1.41–1.96) times increased odds of excess mortality compared to the expected mortality, respectively. Men in the 40–79 years age group had two times higher odds of excess mortality. Men in the 60–69 years age group had a 3.23 (95% CI, 3.15-3.31) times increased odds of excess mortality, while women had a 2.23 (95% CI, 2.16-2.29) times increased odds (Figure 3A,B).

Excess death and excess death rates by sex and regions

The excess all-cause death rate relative to the expected death rate varied between regions. For men, the excess death rate varied from 34% in Cusco to 199% in Lima, while for women, it varied from 7% in Tacna and 125% in Lima. The five regions with the highest excess death rates in men were Lima (199%), Callao (143%), Piura (137%), Moquegua (129%), and Arequipa (123%); the five regions with the highest excess deaths in women were Lima (125%), Callao (94%), Paso (91%), Tumbes (83%), and Piura (78%) (Figure 4A). The highest excess death rates in men (per 100,000 men) and women (per 100,000 women) occurred in Lima, Callao, Moquegua, Arequipa, Ica, and Tumbes (Figure 4B).

ORs for excess death rates stratified by sex and regions

In Figure 5, our meta-analysis shows a continuous increase in the rate of excess deaths in men and women. Men in Lima had 3.01 (95% CI, 2.96–3.06) times increased odds of excess mortality. Men in Callao, Piura, Arequipa, Loreto, Tumbes, and Moquegua had two times increased odds of excess mortality compared to the expected mortality (Figure 5A), while only in Lima did women have a 2.26 (95% CI, 2.21–2.30) times increased odds of excess mortality compared to the expected mortality (Figure 5B).

Temporal distribution of excess deaths by sex and region

Figure 6 shows that the rates of excess deaths increased at the beginning of March and reached a peak at the end of the months of May and August. Some regions showed staggering increases, with rates of excess deaths reaching 500% in men in Loreto in week 18. The rates of excess deaths decreased by week 52 (Figure 7).

For peer review only - http://bmjopen.bmj.com/site/about/guidelines.xhtml

Discussion

During the COVID-19 pandemic. Peru experienced one of the highest excess all-cause mortality rates in the world, along with Bulgaria, North Macedonia, and Serbia.[19] Excess all-cause mortality is recognized as a robust and comparable indicator of mortality associated with the COVID-19 pandemic.[20] Some countries have reported that approximately two thirds of their excess deaths were reported as COVID-19-related deaths.[21] However, in countries such as Peru, where the effects of the pandemic have exceeded the reporting systems for mortality related to COVID-19, the measurement of excess mortality can help guide public health decisions and actions.

According to our analysis, men in Peru had an excess mortality rate almost double that of women. This differs from the data reported in most industrialized and high-income countries where no great difference is evident. For example, the excess mortality in Peruvian men (117%) was double that reported for England and Wales (63%), which, together with Spain, were the countries that experienced the highest excess mortality in Europe;[20,22] this is also higher than the rate reported in other countries of the American continent, such as Mexico (51%) and Brazil (61%).[8,23] Excess mortality in men is likely related to the COVID-19 pandemic, as SARS-CoV-2 has a greater transmissibility in men, which is due mainly to their social and cultural behaviors. It has also been proposed that women have a better protective factor immune response due to their higher number of B lymphocytes and the higher intensity of their inflammatory response against viral diseases.[24,25]

Several previous studies have reported age specific excess deaths. In these studies, there is a wide variability in the estimation of excess mortality by sex and age.[8,26,27] As in Peru, other countries have also reported that excess in observed mortality in younger age groups was similar to at or lower than expected levels.[26] This due to the lower number of unintentional injuries occurring due to COVID-19 containment measures, such as occupational or automobile accidents, the latter being the main cause of years of life lost due to premature death.[28,29] In our study, the excess deaths (%) varied markedly in age groups 50-59, 60-69 and 70-79 years. The rate of excess deaths also varied with aged, and was higher in men aged 60-69, 70-79 and ≥80 years, in contrast to other European countries as Belgium, the Czech Republic, Hungary, Poland, and Scotland where the excess death rates were higher in men of 55-64 years.[26] As in Peru, In overall population in US the excess death in individuals of 65 or more years were higher.[27] In our study, the OR for excess mortality occurred in a wider range of age groups for men (40–79 years old); this may be related to the high prevalence of chronic diseases in adult men, which increases the likelihood of death due to COVID-19.[30,31] Thus, a recent study showed that as obesity prevalence increases, the COVID-19 mortality rates increase in the Peruvian population ≥15 years.[32] These factors may have contributed to a higher estimated excess all-cause deaths rate in men than women in the ≥60 year age in Peru.

Overall, the excess mortality seen in Peru had an asynchronous temporal and geographical distribution. However, men were found to be twice as likely to die in more regions than women were. Regions along the coast (Lima, Callao, Piura, Tumbes), in the Andes (Arequipa, Moquegua), and in the Amazon (Loreto) have large cities with high levels of commercial, migratory, and tourist exchange. In these regions with large cities, the rates of excess deaths in men increased at the beginning of March and reached peak in the weeks 18 and 32, and then decreasing. This increase in all-cause deaths in these regions was temporal, and concomitant with the first wave the COVID-19 pandemic in Peru. The temporal distribution was similar in women (Figure 7A-B). In contrast, the regions of the Andes located at higher altitudes had lower mortality rates. This has been related to the physiological adaptation of this population to a hypoxic environment, which may have protected them from severe impacts of the acute infection caused by SARS-CoV-2; in addition, these areas have a lower prevalence of

For peer review only - http://bmjopen.bmj.com/site/about/guidelines.xhtml

diabetes, obesity, and hypertension.[33] Therefore, the excess all-cause deaths are likely to be a direct effect of the COVID-19 pandemic. These characteristics, together with the low compliance with social distancing measures, the closure of economic activities during the pandemic, the entry of new variants of SARS-CoV-2 with a greater transmissibility, the difficulty of early diagnosis, the collapse of hospitals, and the shortage of medical oxygen could explain the higher excess mortality in Peru.[34,35] The changes in population (male and female) behavior brought about by lockdown measures could also have had effects on excess deaths and the mortality rate. Finally, during the country-wide lockdown implemented in Peru, access to healthcare was limited and many medical procedures were delayed. Therefore, the effects of the lockdown may be another explanation for the excess number of deaths and excess death rate.

Weaknesses of this study

Our study has several important limitations. First, although SINADEF is an official database, there may have been delays in its registration due to a lack of notification of non-hospital or manually registered deaths. Therefore, the number of excess all-cause deaths registered might have been underestimated.

A second limitation is related to the selection of expected deaths, a study of excess mortality during the pandemic carried out in 103 countries mentions that 20 of them, in which Peru is located, have a death record of less than 90% according to the Demographic Yearbook of the United Nations for 2019,[19] although there was a trend in the increase in the coverage of registered deaths according to data from 2012 (70%) to 2016 (80%), it is in 2017 that improvements to the SINADEF of Peru, with the collaboration of international institutions and that have allowed the increase in the use of death certificates online, and the improvement in the quality of the information, through training, standardization and monitoring of civil registration and vital statistics processes, shortening the gap between the coverage of registered and estimated deaths.[36] However, it is likely that an increase in the registration of deaths expected during 2017-2019 is related to the coverage of deaths, therefore the use of an average calculated in epidemiological weeks would be justified.

Third, the analyzes could limit its comparison with other studies, where excess mortality uses complex models that apply periodic splines or Fourier harmonics to smooth and homogenize possible fluctuations in expected deaths and standardize their measurements.[35] We using P-score, a standard measure, simple and easy to replicate in any context, similar to that carried out in study others;[8,27] however, it was only possible to access three years as a baseline for expected deaths in 2020 (based on the years 2017–2019). This method could also be easily replicated in each region without losing the opportunity to analyze variables important;[8] however, it was only possible to access three years as a baseline. Future analyzes of excess deaths in 2021 could not include 2020 in the average expected deaths due to high death rates.

Strengths of this study

The main strength of our study is that the method developed allows the transparency and reproducibility of the findings. The baseline for expected deaths made it possible to capture the weekly variation of observed deaths and is self-consistent with that reported in more complex methodologies.[37] The simplicity in the analysis of excess mortality allows improving the opportunity to use the findings in epidemiological surveillance and their interpretation by those responsible for the formulation of public policies and health authorities at different levels.

Implications for policymakers

For peer review only - http://bmjopen.bmj.com/site/about/guidelines.xhtml

The findings add evidence to the understanding of excess mortality in Peru. Although the initiation of vaccination against COVID-19, and the improvements in the capacity to diagnose and care for critical patients, will reduce mortality, policymakers must evaluate future measurements and interventions based on gender. On the other hand, coordination initiatives between those responsible for epidemiological surveillance, vital event registration systems, and diagnosis have contributed to obtaining more realistic numbers, but evaluating the interoperability of computer and statistical systems is necessary for future measurements of observed deaths and excess mortality. Finally, having up-to-date information provides government and public health officials with adequate mechanisms to track the impact of the pandemic.

Conclusion

Approximately one hundred thousand excess deaths occurred in men and women during the COVID-19 pandemic in Peru. The age-stratified excess death rates were higher in men than in women, and men between the ages of 40 and 79 years experienced twice the rate of excess deaths compared with the rate expected. Strong excesses in geographical and temporal mortality patterns by region were found in both men and women. These findings reveal the impact of the COVID-19 pandemic on all-cause mortality up to the point where vaccination against SARS-CoV-2 started to become available in Peru.

Footnotes

Contributors. MCRS designed the study and oversaw the acquisition of data. MCRS, GOC, HAH performed the data collection and interpretation of the data. MCRS and HAH verified the coded results. MCRS and HAH analysed the data. MCRS, GOC, HAH contributed to drafting the manuscript. MCRS, GOC, HAH were involved in critical revisions of the manuscript for important intellectual content. All authors approved the final draft of the manuscript.

Funding. This study has no funding.

Competing. The authors declare no conflict of interest.

Ethics statements. Patient consent for publication. Not required.

Data availability statement. The data presented in this study are publicly available at:

SINADEF, MINSA: https://www.datosabiertos.gob.pe/dataset/fallecidos-por-covid-19-ministerio-de-salud-minsa

INEI, Peruvian population: https://www.inei.gob.pe/estadisticas/indice-tematico/population-estimates-and-projections/

Acknowledgments. Not available

References

1. Dhama K, Khan S, Tiwari R, Sircar S, Bhat S, Malik YS, et al. Coronavirus Disease 2019-COVID-19. Clin Microbiol Rev. 2020;33(4):e00028-20.
2. O'Driscoll M, Ribeiro Dos Santos G, Wang L, Cummings DAT, Azman AS, Paireau J, et al. Age-specific mortality and immunity patterns of SARS-CoV-2. Nature. 2021;590(7844):140-145.

For peer review only - http://bmjopen.bmj.com/site/about/guidelines.xhtml

3. Bilinski A, Emanuel EJ. COVID-19 and Excess All-Cause Mortality in the US and 18 Comparison Countries. JAMA. 2020;324(20):2100-2102.
4. Chen YH, Glymour MM, Catalano R, Fernandez A, Nguyen T, Kushel M, et al. Excess Mortality in California During the Coronavirus Disease 2019 Pandemic, March to August 2020. JAMA Intern Med. 2021 May 1;181(5):705-707.
5. Banerjee A, Pasea L, Harris S, Gonzalez-Izquierdo A, Torralbo A, Shallcross L, et al. Estimating excess 1-year mortality associated with the COVID-19 pandemic according to underlying conditions and age: a population-based cohort study. Lancet. 2020;395(10238):1715-1725.
6. Kontopantelis E, Mamas MA, Deanfield J, Asaria M, Doran T. Excess mortality in England and Wales during the first wave of the COVID-19 pandemic. J Epidemiol Community Health. 2021;75(3):213-223.
7. Gianicolo EAL, Russo A, Büchler B, Taylor K, Stang A, Blettner M. Gender specific excess mortality in Italy during the COVID-19 pandemic accounting for age. Eur J Epidemiol. 2021 Feb;36(2):213-218.
8. Palacio-Mejía LS, Wheatley-Fernández JL, Ordóñez-Hernández I, López-Ridaura R, López Gatell-Ramírez H, Hernández-Ávila M, et al. All-cause excess mortality during the Covid-19 pandemic in Mexico. Salud Publica Mex. 2021;63:211-224
9. Locatelli I, Rousson V. A first analysis of excess mortality in Switzerland in 2020. PLoS ONE, 2021;16(6): e0253505
10. Liu J, Zhang L, Yan Y, Zhou Y, Yin P, Qi J, et al. Excess mortality in Wuhan city and other parts of China during the three months of the covid-19 outbreak: findings from nationwide mortality registries. BMJ. 2021;372:n415.
11. Islam N, Shkolnikov VM, Acosta RJ, Klimkin I, Kawachi I, Irizarry RA, et al. Excess deaths associated with covid-19 pandemic in 2020: age and sex disaggregated time series analysis in 29 high income countries. BMJ. 2021;373:n1137.
12. Peruvian Ministry of Health (MINSA). Sala Situaional. COVID-19 Peru. Lima: MINSA; 2021 (accessed on 27 July 2021). Available online: https://covid19.minsa.gob.pe/
13. Peruvian Ministry of Health (MINSA). Excess Mortality. Lima: MINSA; Julay 25, 2021. Available in: https://www.minsa.gob.pe/reunis/data/exceso_mortalidad.asp
14. Ramírez-Soto MC, Arroyo-Hernández H, Ortega-Cáceres G (2021) Sex differences in the incidence, mortality, and fatality of COVID-19 in Peru. PLoS ONE 16(6): e0253193.
15. Munayco C, Chowell G, Tariq A, Undurraga EA, Mizumoto K. Risk of death by age and gender from CoVID-19 in Peru, March-May, 2020. Aging (Albany NY). 2020;12(14):13869–81.
16. Vandenbroucke JP, von Elm E, Altman DG, Gøtzsche PC, Mulrow CD, Pocock SJ, Poole C, Schlesselman JJ, Egger M; STROBE Initiative. Strengthening the Reporting of Observational Studies in Epidemiology (STROBE): explanation and elaboration. PLoS Med. 2007 Oct 16;4(10):e297.
17. Peruvian Ministry of Health (MINSA). COVID-19 deaths. National System of Deaths (SINADEF). Lima: MINSA; 2021 (accessed on 03 June 2021). Available online: https://www.datosabiertos.gob.pe/dataset/fallecidos-por-covid-19-ministerio-de-salud-minsa
18. National Institute of Statistics and Informatics (INEI). Peruvian Population. Available online: https://www.inei.gob.pe/estadisticas/indice-tematico/population-estimates-and-projections/ (accessed on 3 June 2021).
19. Karlinsky A, Kobak D. Tracking excess mortality across countries during the COVID-19 pandemic with the World Mortality Dataset. Elife. 2021;10:e69336.

For peer review only - http://bmjopen.bmj.com/site/about/guidelines.xhtml

20. Vestergaard LS, Mølbak K. Timely monitoring of total mortality associated with COVID-19: informing public health and the public. Euro Surveill. 2020;25(34):2001591.
21. Woolf SH, Chapman DA, Sabo RT, Weinberger DM, Hill L, Taylor DDH. Excess Deaths From COVID-19 and Other Causes, March-July 2020. *JAMA* 2020;324:1562-4. Doi:10.1001/jama.2020.19545
22. Kontis V, Bennett JE, Rashid T, Parks RM, Pearson-Stuttard J, Guillot M, *et al.* Magnitude, demographics and dynamics of the effect of the first wave of the Covid-19 pandemic on all-cause mortality in 21 industrialized countries. Nat Med. 2020;26:1919-28.
23. Silva GAE, Jardim BC, Santos C. Excess mortality in Brazil in times of Covid-19. Cien Saude Colet. 2020;25(9):3345-54.
24. Gebhard C, Regitz-Zagrosek V, Neuhauser HK, Morgan R, Klein SL. Impact of sex and gender on COVID-19 outcomes in Europe. Biol Sex Differ. 2020; 11(1):29. 11:29. pmid:32450906
25. Abate BB, Kassie AM, Kassaw MW, Aragie TG, Masresha SA. Sex difference in coronavirus disease (COVID-19): a systematic review and meta-analysis. BMJ Open. 2020 Oct 6;10(10):e040129.
26. Islam N, Shkolnikov VM, Acosta RJ, Klimkin I, Kawachi I, Irizarry RA. Excess deaths associated with covid-19 pandemic in 2020: age and sex disaggregated time series analysis in 29 high income countries. BMJ 2021;373:n1137.
27. Aron J, Muellbauer J. A Pandemic Primer on Excess Mortality Statistics and Their Comparability Across Countries. Our World in Data; 2020. Available in: https://ourworldindata.org/covid-excess-mortality
28. Velásquez A. La carga de enfermedad y lesiones en el Perú y las prioridades del plan esencial de aseguramiento universal. Rev Perú Med Exp Salud Publica. 2009; 26(2): 222-231.
29. Calderon-Anyosa RJC, Kaufman JS. Impact of COVID-19 lockdown policy on homicide, suicide, and motor vehicle deaths in Peru. Prev Med. 2021;143:106331
30. Yamada G, Castillo-Salgado C, Jones-Smith JC, Moulton LH. Differences in magnitude and rate of change in adult obesity distribution by age and sex in Mexico, Colombia and Peru, 2005-2010. Public Health Nutr. 2019 Mar;22(4):757-763.
31. Zhou F, Yu T, Du R, et al. Clinical course and risk factors for mortality of adult inpatients with COVID-19 in Wuhan, China: a retrospective cohort study. Lancet. 2020;395(10229):1054–1062.
32. Ramírez-Soto MC, Alarcón-Arroyo M, Chilcon-Vitor Y, Chirinos-Pérez Y, Quispe-Vargas G, Solsol-Jacome K, et al. Association between Obesity and COVID-19 Mortality in Peru: An Ecological Study. Tropical Medicine and Infectious Disease. 2021;6(4):182.
33. Seclén SN, Nunez-Robles E, Yovera-Aldana M, Arias-Chumpitaz A. Incidence of COVID-19 infection and prevalence of diabetes, obesity and hypertension according to altitude in Peruvian population. Diabetes Res Clin Pract. 2020 Nov;169:108463.
34. Álvarez-Antonio C, Meza-Sánchez G, Calampa C, Casanova W, Carey C, Alava F. et al. Seroprevalence of anti-SARS-CoV-2 antibodies in Iquitos, Peru in July and August, 2020: a population-based study. Lancet Glob Health. 2021 Jul;9(7):e925-e931.
35. Reyes-Vega MF, Soto-Cabezas MG, Cárdenas F, Martel KS, Valle A, Valverde J. et al. SARS-CoV-2 prevalence associated to low socioeconomic status and overcrowding in an LMIC megacity: A population-based seroepidemiological survey in Lima, Peru. EClinicalMedicine. 2021;34:100801.

For peer review only - http://bmjopen.bmj.com/site/about/guidelines.xhtml

36. Vargas-Herrera J, Pardo-Ruiz K, Garro-Nuñez G, Miki-Ohno J, Pérez-Lu JE, Valdez-Huarcaya W, et al. [Preliminary results of the strengthening of the national death registry information system]. Rev Peru Med Exp Salud Publica. 2018;35(3):505–514. (in Spanish)

37. Sempé L, Lloyd-Sherlock P, Martínez R, Ebrahim S, McKee M, Acosta E. Estimation of all-cause excess mortality by age-specific mortality patterns for countries with incomplete vital statistics: a population-based study of the case of Peru during the first wave of the COVID-19 pandemic. Lancet Reg Health Am. 2021.

Legends:

Figure 1. The study flow chart of all-cause death excess with the STROBE (Strengthening the Reporting of Observational Studies in Epidemiology)

Figure 2. Excess numbers of deaths (A) and excess death rates (B) in men and women in Peru stratified by age.

Figure 3. ORs for excess death rates stratified by age in men (A) and women (B) in Peru.

Figure 4. Observed, expected, and excess death (A) and excess death rates (B) stratified by region in men and women in Peru.

Figure 5. ORs for excess death rates according to region in men (A) and women (B) in Peru.

Figure 6. Temporal distribution of excess deaths (%) in men and women in Peru.

Figure 7. Temporal distribution of excess deaths according to region in men (A) and women (B) in Peru.

For peer review only

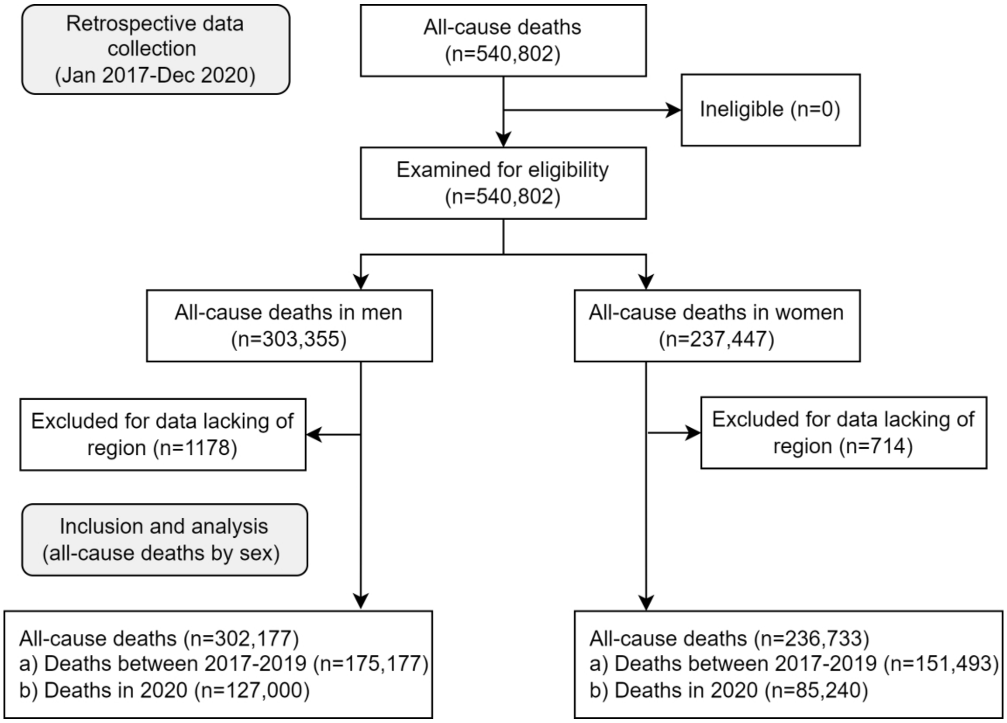

Figure 1. The study flow chart of all-cause death excess with the STROBE (Strengthening the Reporting of Observational Studies in Epidemiology)

189x135mm (300 x 300 DPI)

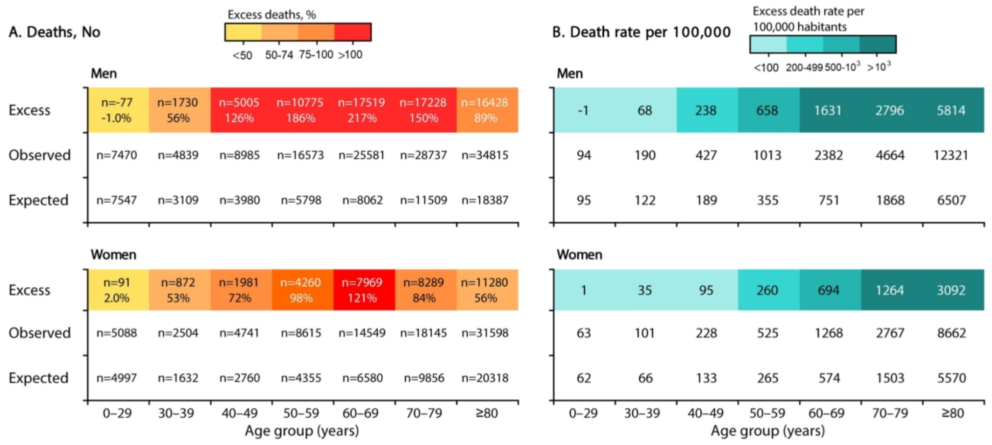

Figure 2. Excess numbers of deaths (A) and excess death rates (B) in men and women in Peru stratified by age.

190x86mm (300 x 300 DPI)

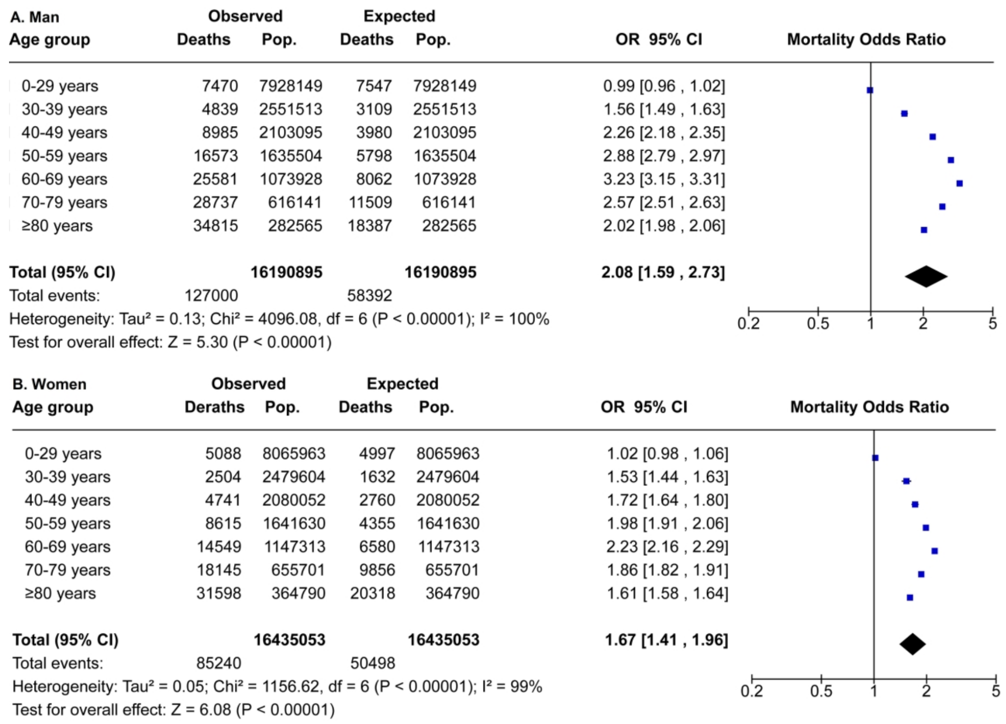

Figure 3. ORs for excess death rates stratified by age in men (A) and women (B) in Peru.

190x137mm (300 x 300 DPI)

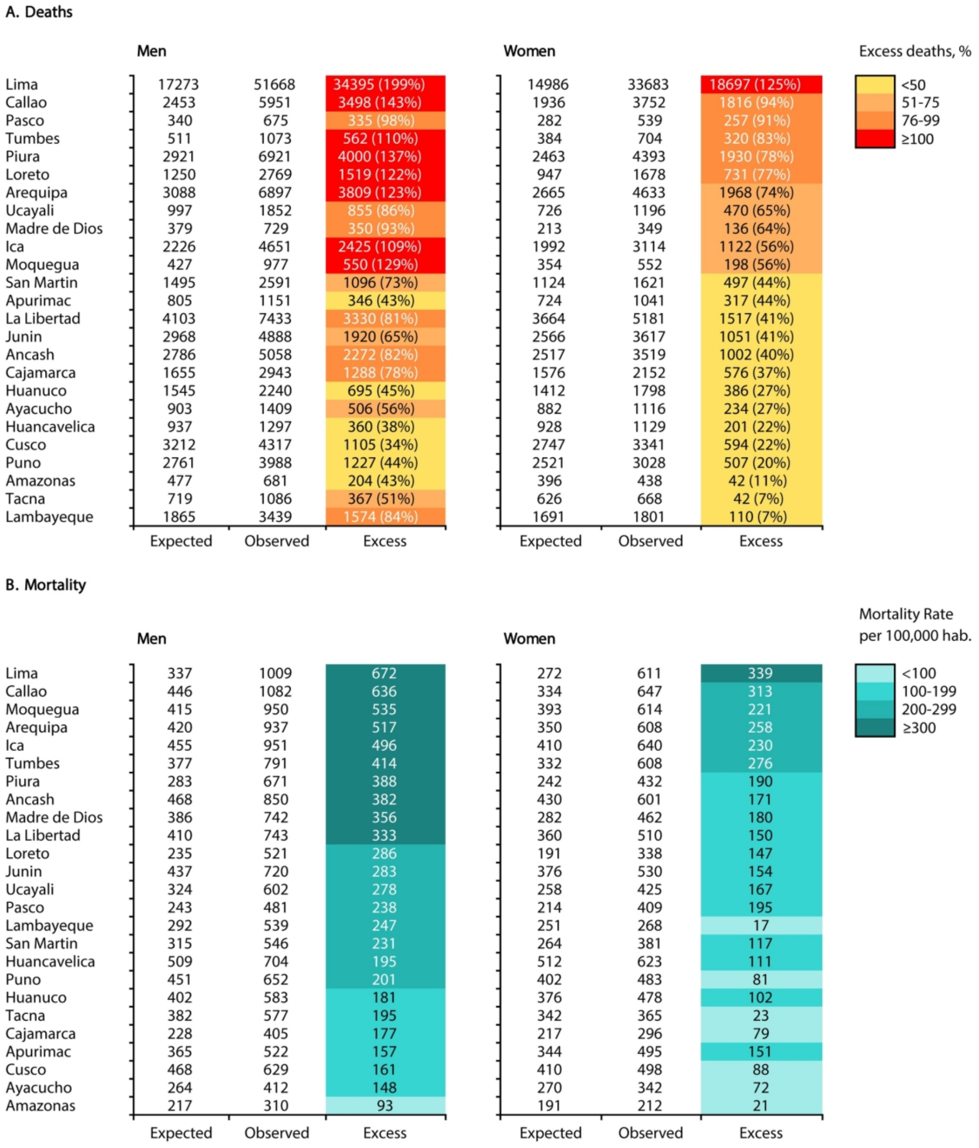

Figure 4. Observed, expected, and excess death (A) and excess death rates (B) stratified by region in men and women in Peru.

181x216mm (300 x 300 DPI)

A. Men<br>Region	Observed<br>Deaths	 Pop.	Expected<br>Deaths	 Pop.	OR 95% CI
Amazonas	681	219801	477	219801	1.43 [1.27 , 1.61]
Ancash	5058	594832	2786	594832	1.82 [1.74 , 1.91]
Apurimac	1151	220370	805	220370	1.43 [1.31 , 1.57]
Arequipa	6897	735707	3088	735707	2.25 [2.15 , 2.34]
Ayacucho	1409	341951	903	341951	1.56 [1.44 , 1.70]
Cajamarca	2943	727265	1655	727265	1.78 [1.68 , 1.89]
Callao Province	5951	550046	2453	550046	2.44 [2.33 , 2.56]
Cusco	4317	686543	3212	686543	1.35 [1.29 , 1.41]
Huancavelica	1297	184121	937	184121	1.39 [1.27 , 1.51]
Huanuco	2240	384345	1545	384345	1.45 [1.36 , 1.55]
Ica	4651	488836	2226	488836	2.10 [2.00 , 2.21]
Junin	4888	678494	2968	678494	1.65 [1.58 , 1.73]
La Libertad	7433	1000002	4103	1000002	1.82 [1.75 , 1.89]
Lambayeque	3439	638228	1865	638228	1.85 [1.75 , 1.96]
Lima	51668	5119560	17273	5119560	3.01 [2.96 , 3.06]
Loreto	2769	531000	1250	531000	2.22 [2.08 , 2.38]
Madre de Dios	729	98215	379	98215	1.93 [1.70 , 2.19]
Moquegua	977	102855	427	102855	2.30 [2.05 , 2.58]
Pasco	675	140252	340	140252	1.99 [1.75 , 2.27]
Piura	6921	1030975	2921	1030975	2.38 [2.28 , 2.48]
Puno	3988	611616	2761	611616	1.45 [1.38 , 1.52]
San Martin	2591	474458	1495	474458	1.74 [1.63 , 1.85]
Tacna	1086	188152	719	188152	1.51 [1.38 , 1.66]
Tumbes	1073	135675	511	135675	2.11 [1.90 , 2.34]
Ucayali	1852	307596	997	307596	1.86 [1.72 , 2.01]
**Total (95% CI)**		**16190895**		**16190895**	**1.83 [1.63 , 2.06]**
Total events:	126684		58096		

Heterogeneity: Tau² = 0.09; Chi² = 3033.65, df = 24 (P < 0.00001); I² = 99%

Test for overall effect: Z = 10.06 (P < 0.00001)

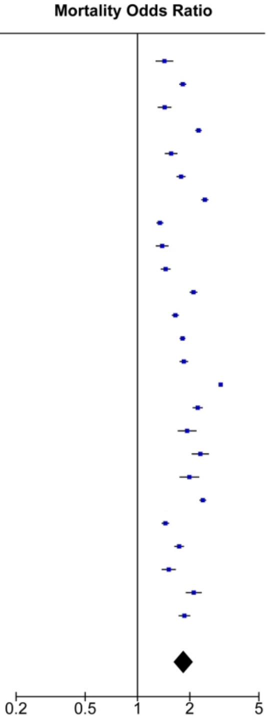

Figure 5A. ORs for excess death rates according to region in men in Peru

190x147mm (300 x 300 DPI)

B. Women	Observed		Expected			
**Region**	**Deaths**	**Pop.**	**Deaths**	**Pop.**	**OR 95% CI**	**Mortality Odds Ratio**
Amazonas	438	207005	396	207005	1.11 [0.97 , 1.27]	
Ancash	3519	585806	2517	585806	1.40 [1.33 , 1.47]	
Apurimac	1041	210366	724	210366	1.44 [1.31 , 1.58]	
Arequipa	4633	761731	2665	761731	1.74 [1.66 , 1.83]	
Ayacucho	1116	326262	882	326262	1.27 [1.16 , 1.38]	
Cajamarca	2152	727265	1576	727265	1.37 [1.28 , 1.46]	
Callao Province	3752	579808	1936	579808	1.94 [1.84 , 2.05]	
Cusco	3341	670532	2747	670532	1.22 [1.16 , 1.28]	
Huancavelica	1129	181196	928	181196	1.22 [1.12 , 1.33]	
Huanuco	1798	375922	1412	375922	1.27 [1.19 , 1.37]	
Ica	3114	486346	1992	486346	1.57 [1.48 , 1.66]	
Junin	3617	682973	2566	682973	1.41 [1.34 , 1.49]	
La Libertad	5181	1016769	3664	1016769	1.42 [1.36 , 1.48]	
Lambayeque	1801	672557	1691	672557	1.07 [1.00 , 1.14]	
Lima	33683	5508910	14986	5508910	2.26 [2.21 , 2.30]	
Loreto	1678	496559	947	496559	1.77 [1.64 , 1.92]	
Madre de Dios	349	75596	213	75596	1.64 [1.38 , 1.95]	
Moquegua	552	89885	354	89885	1.56 [1.37 , 1.79]	
Pasco	539	131652	282	131652	1.92 [1.66 , 2.21]	
Piura	4393	1016979	2463	1016979	1.79 [1.70 , 1.88]	
Puno	3028	626381	2521	626381	1.20 [1.14 , 1.27]	
San Martin	1621	425190	1124	425190	1.44 [1.34 , 1.56]	
Tacna	668	182822	626	182822	1.07 [0.96 , 1.19]	
Tumbes	704	115846	384	115846	1.84 [1.62 , 2.08]	
Ucayali	1196	281514	726	281514	1.65 [1.50 , 1.81]	
**Total (95% CI)**		**16435872**		**16435872**	**1.47 [1.33 , 1.64]**	
Total events:	85043		50322			

Heterogeneity: Tau² = 0.07; Chi² = 1911.23, df = 24 (P < 0.00001); I² = 99%
Test for overall effect: Z = 7.20 (P < 0.00001)

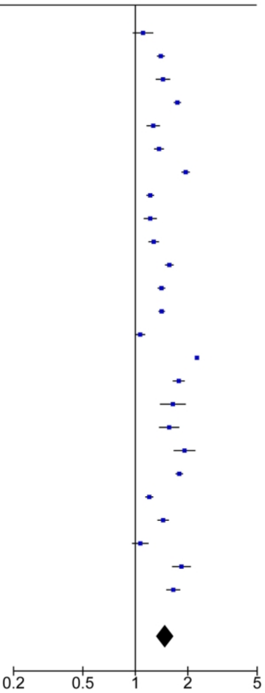

Figure 5B. ORs for excess death rates according to region in women in Peru

190x146mm (300 x 300 DPI)

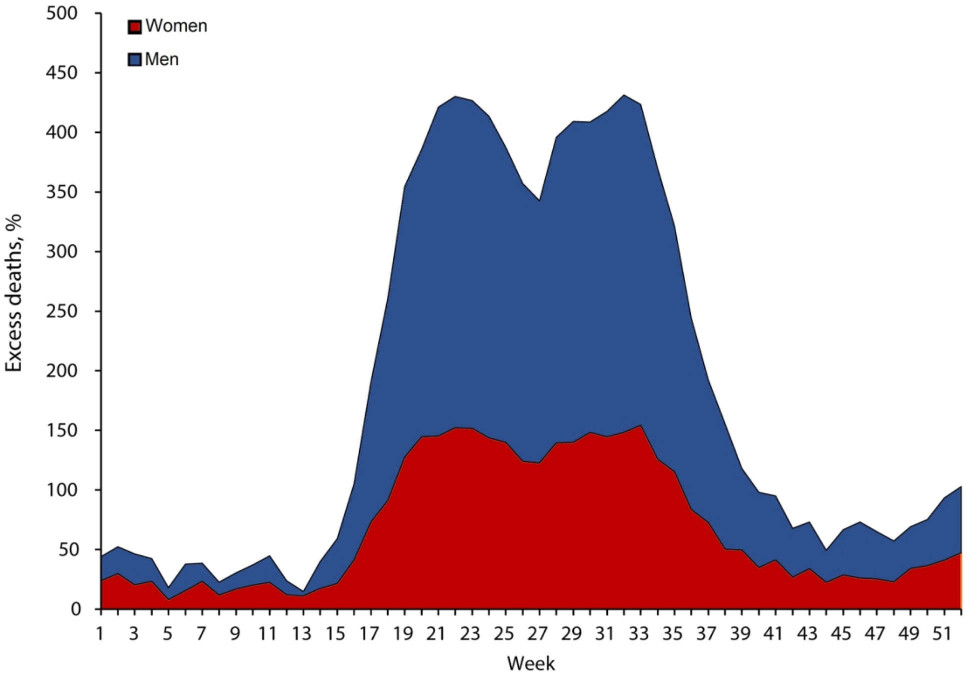

Figure 6. Temporal distribution of excess deaths (%) in men and women in Peru.

154x110mm (300 x 300 DPI)

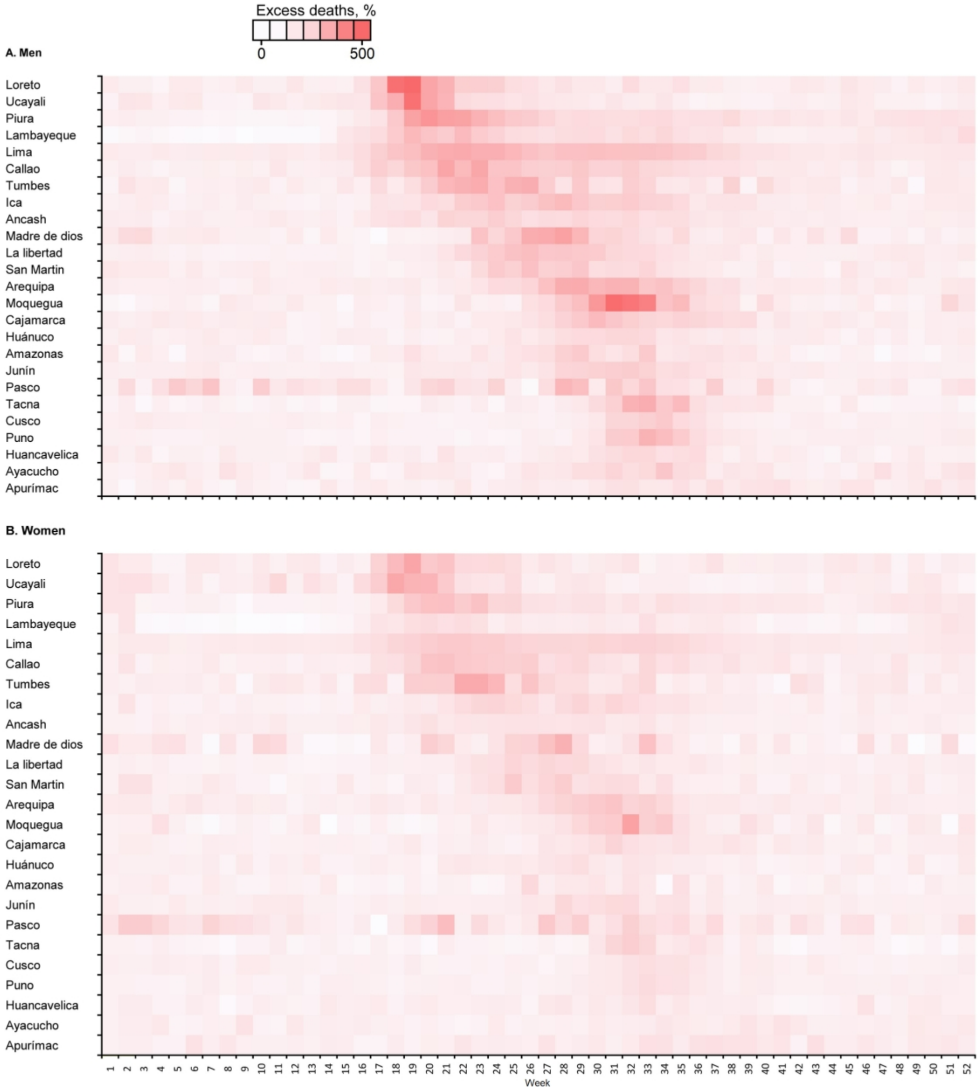

Figure 7. Temporal distribution of excess deaths according to region in men (A) and women (B) in Peru

181x198mm (300 x 300 DPI)

STROBE Statement—checklist of items that should be included in reports of observational studies

	Item No	Recommendation	Page No
**Title and abstract**	1	(*a*) Indicate the study's design with a commonly used term in the title or the abstract	1
		(*b*) Provide in the abstract an informative and balanced summary of what was done and what was found	2
**Introduction**			
Background/rationale	2	Explain the scientific background and rationale for the investigation being reported	4
Objectives	3	State specific objectives, including any prespecified hypotheses	4
**Methods**			
Study design	4	Present key elements of study design early in the paper	4
Setting	5	Describe the setting, locations, and relevant dates, including periods of recruitment, exposure, follow-up, and data collection	5
Participants	6	(*a*) *Cohort study*—Give the eligibility criteria, and the sources and methods of selection of participants. Describe methods of follow-up<br>*Case-control study*—Give the eligibility criteria, and the sources and methods of case ascertainment and control selection. Give the rationale for the choice of cases and controls<br>*Cross-sectional study*—Give the eligibility criteria, and the sources and methods of selection of participants	Fig. 1
		(*b*) *Cohort study*—For matched studies, give matching criteria and number of exposed and unexposed<br>*Case-control study*—For matched studies, give matching criteria and the number of controls per case	NA
Variables	7	Clearly define all outcomes, exposures, predictors, potential confounders, and effect modifiers. Give diagnostic criteria, if applicable	5
Data sources/ measurement	8*	For each variable of interest, give sources of data and details of methods of assessment (measurement). Describe comparability of assessment methods if there is more than one group	*5*
Bias	9	Describe any efforts to address potential sources of bias	5
Study size	10	Explain how the study size was arrived at	5

For peer review only

Quantitative variables	11	Explain how quantitative variables were handled in the analyses. If applicable, describe which groupings were chosen and why	5
Statistical methods	12	(*a*) Describe all statistical methods, including those used to control for confounding	5
		(*b*) Describe any methods used to examine subgroups and interactions	5
		(*c*) Explain how missing data were addressed	5
		(*d*) *Cohort study*—If applicable, explain how loss to follow-up was addressed<br>*Case-control study*—If applicable, explain how matching of cases and controls was addressed<br>*Cross-sectional study*—If applicable, describe analytical methods taking account of sampling strategy	NA
		(*e*) Describe any sensitivity analyses	NA

Continued on next page

For peer review only

**Results**			
Participants	13*	(a) Report numbers of individuals at each stage of study—eg numbers potentially eligible, examined for eligibility, confirmed eligible, included in the study, completing follow-up, and analysed	5
		(b) Give reasons for non-participation at each stage	5
		(c) Consider use of a flow diagram	5
Descriptive data	14*	(a) Give characteristics of study participants (eg demographic, clinical, social) and information on exposures and potential confounders	5
		(b) Indicate number of participants with missing data for each variable of interest	5
		(c) *Cohort study*—Summarise follow-up time (eg, average and total amount)	NA
Outcome data	15*	*Cohort study*—Report numbers of outcome events or summary measures over time	*NA*
		Case-control study—Report numbers in each exposure category, or summary measures of exposure	*NA*
		Cross-sectional study—Report numbers of outcome events or summary measures	*NA*
Main results	16	(*a*) Give unadjusted estimates and, if applicable, confounder-adjusted estimates and their precision (eg, 95% confidence interval). Make clear which confounders were adjusted for and why they were included	6
		(*b*) Report category boundaries when continuous variables were categorized	6
		(*c*) If relevant, consider translating estimates of relative risk into absolute risk for a meaningful time period	6
Other analyses	17	Report other analyses done—eg analyses of subgroups and interactions, and sensitivity analyses	6
**Discussion**			
Key results	18	Summarise key results with reference to study objectives	7
Limitations	19	Discuss limitations of the study, taking into account sources of potential bias or imprecision. Discuss both direction and magnitude of any potential bias	7
Interpretation	20	Give a cautious overall interpretation of results considering objectives, limitations, multiplicity of analyses, results from similar studies, and other relevant evidence	8-9
Generalisability	21	Discuss the generalisability (external validity) of the study results	8-9
**Other information**			

For peer review only

Funding	22	Give the source of funding and the role of the funders for the present study and, if applicable, for the original study on which the present article is based	9

*Give information separately for cases and controls in case-control studies and, if applicable, for exposed and unexposed groups in cohort and cross-sectional studies.

Note: An Explanation and Elaboration article discusses each checklist item and gives methodological background and published examples of transparent reporting. The STROBE checklist is best used in conjunction with this article (freely available on the Web sites of PLoS Medicine at http://www.plosmedicine.org/, Annals of Internal Medicine at http://www.annals.org/, and Epidemiology at http://www.epidem.com/). Information on the STROBE Initiative is available at www.strobe-statement.org.

NA: Not available

For peer review only

BMJ Open

Excess all-cause deaths stratified by sex and age in Peru: a time series analysis during the COVID-19 pandemic

Journal:	*BMJ Open*
Manuscript ID	bmjopen-2021-057056.R2
Article Type:	Original research
Date Submitted by the Author:	26-Jan-2022
Complete List of Authors:	Ramírez-Soto, Max Carlos; Universidad San Martin de Porres, Centro de Investigación en Salud Publica, Facultad de Medicina Humana; Universidad Tecnológica del Perú<br>Ortega-Cáceres, Gutia; Universidad Ricardo Palma<br>Arroyo-Hernández, Hugo; Instituto Nacional de Salud
**Primary Subject Heading**:	Epidemiology
Secondary Subject Heading:	Epidemiology, Health policy
Keywords:	COVID-19, EPIDEMIOLOGY, PUBLIC HEALTH, INFECTIOUS DISEASES

SCHOLARONE™
Manuscripts

I, the Submitting Author has the right to grant and does grant on behalf of all authors of the Work (as defined in the below author licence), an exclusive licence and/or a non-exclusive licence for contributions from authors who are: i) UK Crown employees; ii) where BMJ has agreed a CC-BY licence shall apply, and/or iii) in accordance with the terms applicable for US Federal Government officers or employees acting as part of their official duties; on a worldwide, perpetual, irrevocable, royalty-free basis to BMJ Publishing Group Ltd ("BMJ") its licensees and where the relevant Journal is co-owned by BMJ to the co-owners of the Journal, to publish the Work in this journal and any other BMJ products and to exploit all rights, as set out in our licence.

The Submitting Author accepts and understands that any supply made under these terms is made by BMJ to the Submitting Author unless you are acting as an employee on behalf of your employer or a postgraduate student of an affiliated institution which is paying any applicable article publishing charge ("APC") for Open Access articles. Where the Submitting Author wishes to make the Work available on an Open Access basis (and intends to pay the relevant APC), the terms of reuse of such Open Access shall be governed by a Creative Commons licence – details of these licences and which Creative Commons licence will apply to this Work are set out in our licence referred to above.

Other than as permitted in any relevant BMJ Author's Self Archiving Policies, I confirm this Work has not been accepted for publication elsewhere, is not being considered for publication elsewhere and does not duplicate material already published. I confirm all authors consent to publication of this Work and authorise the granting of this licence.

For peer review only

Excess all-cause deaths stratified by sex and age in Peru: a time series analysis during the COVID-19 pandemic

Max Carlos Ramírez-Soto [1,2,*], Gutia Ortega-Cáceres [3], Hugo Arroyo-Hernández [4]

[1] Centro de Investigacion en Salud Publica, Facultad de Medicina Humana, Universidad San Martin de Porres, Lima, Peru

[2] Facultad de Ciencias de la Salud, Universidad Tecnológica del Peru, Lima, Peru

[3] Escuela de Posgrado, Universidad Ricardo Palma, Lima, Peru

[4] Oficina General de Información y Sistemas, Instituto Nacional de Salud, Lima, Peru

***Correspondence to:**

Max Carlos Ramírez-Soto, BSc, MPH, FRSPH;

maxcrs22@gmail.com

Conflicts of Interest: The authors declare no conflict of interest.

Funding: Please add: “This research received no external funding”

Words: 3101

Figures: 07

For peer review only - http://bmjopen.bmj.com/site/about/guidelines.xhtml

Abstract

Background. In this study, we estimated the excess all-cause deaths and excess death rates during the COVID-19 pandemic in 25 Peruvian regions, stratified by sex and age groups.

Design. Cross-sectional study.

Setting. Twenty-five Peruvian regions with complete mortality data.

Participants. Annual all-cause official mortality data set from the Sistema Informatico Nacional de Defunciones (SINADEF) at the Ministry of Health of Peru for 2017–20, disaggregated by age and sex.

Main outcome measures. Excess deaths and the excess death rates (observed deaths vs. expected deaths) in 2020 by sex and age (0–29, 30–39, 40–49, 50–59, 60–69, 70–79 and ≥80 years) were estimated using P-score. The odds ratios (ORs) for excess mortality were summarized with a random effects meta-analysis.

Results. In the period between January and December 2020, we estimated an excess of 68,608 (117%) deaths in men and 34,742 (69%) deaths in women, corresponding to an excess death rate of 424 per 100,000 men and 211 per 100,000 women compared to the expected mortality rates. The number of excess deaths increased with age and was higher in men aged 60–69 years (217%) compared with women (121%). Men between the ages of 40 and 79 years experienced twice the rate of excess deaths compared with the expected rate. In eight regions, excess deaths were higher than 100% in men, and in seven regions excess deaths were higher than 70% in women. Men in eight regions and women in one region had two times increased odds of excess death than the expected mortality. There were differences in excess mortality according to the temporal distribution by epidemiological week.

Conclusion. Approximately one hundred thousand excess all-cause deaths occurred in 2020 in Peru. The age-stratified excess death rates were higher in men than in women. There were strong excesses in geographical and temporal mortality patterns according to region.

Keywords: excess mortality; sex; mortality rate; COVID-19; Peru

For peer review only

Strengths and limitations of this study

- The main strength of our study was the large number of deaths included (302,177 in men and 236,733 in women) for estimating the excess all-cause deaths.
- Although our analysis was based in proportion of mortality count excess (%) and the excess death rates (per 100,000 inhabitants), our estimates of excess all-cause deaths are similar to estimates of modeled studies.
- The simplicity in the analysis of excess mortality offers an opportunity to use the findings in epidemiological surveillance and their interpretation by those responsible for the formulation of public policies and health authorities at different levels.
- Although SINADEF is an official database, there may have been delays in its registration due to a lack of notification of non-hospital or manually registered deaths.
- We present the results of a descriptive analysis and so we are not able to comment on causality in excess deaths.

For peer review only

For peer review only - http://bmjopen.bmj.com/site/about/guidelines.xhtml

Introduction

Following the outbreak of COVID-19 in China in 2019, SARS-CoV-2 spread rapidly worldwide.[1] During the weeks that followed, there was a rapid increase in the incidence and mortality rates of COVID-19 in European countries, the USA, and Latin American countries.[2] Previous studies on excess deaths (the gap between observed and expected deaths) during the COVID-19 pandemic found that COVID-19-related deaths and deaths from other diseases increased along with age and were higher in men 60 years of age and older compared with women.[3–10] More recently, an assessment of the direct and indirect effects of the COVID-19 pandemic on mortality has revealed that approximately one million excess deaths occurred in 2020 in 29 high-income countries. Additionally, these excess death rates were higher in men than in women in almost all countries.[11]

Peru is one of the countries that has been the most affected by the COVID-19 pandemic. The first cases in Peru were reported in March 2020. In the weeks that followed, rapidly increasing numbers of cases and fatalities were observed across many Peruvian regions. By the end of December 2020, about 1 million confirmed COVID-19 cases and more than 90,000 deaths had been officially reported.[12] In Peru, data on excess all-cause deaths have been reported, but analyses have not accounted for age and sex standardization. From 29 March 2020 (week 14), a significant rate of excess deaths in comparison with the expected deaths based on the figures from 2017 to 2019 have been observed at the national level, with this rate reaching the highest level in week 32 and then decreasing.[13] This increase in all-cause deaths is concomitant with the COVID-19 pandemic, as it has previously been shown that COVID-19 mortality rates increase with age and are high in men aged 60 years and over.[14,15] Despite these findings, to date the excess mortality stratified by sex, age, and region is not known at a national level. The assessment of the impact of the COVID-19 pandemic on mortality in Peru should include the analysis of both the direct effect of the pandemic on deaths caused by COVID-19 and the indirect effect of the pandemic on deaths unrelated to COVID-19, since patients with chronic conditions may have been turned away from the health care system because of concerns relating to COVID-19 infection and social and economic changes.

In this study, we estimated the excess all-cause deaths and the excess death rate during the first year of the COVID-19 pandemic in 25 Peruvian regions, stratified by sex, age, group, and epidemiological week.

Methods

Study design

We performed a cross-sectional study following the Strengthening the Reporting of Observational Studies in Epidemiology (STROBE) reporting guidelines.[16] This study was a time series analysis of annual data on all-cause mortality obtained from 25 Peruvian regions and contained official, valid, and complete mortality data between 2017 and 2020, disaggregated by age and sex. The Peruvian regions are first-level administrative divisions of the country.

Data collection

We used weekly death data extracted from the National Information Technology System for Death Records (in Spanish, 'Sistema Informático Nacional de Defunciones' (SINADEF)) at the Ministry of Health of Peru.[17] The source data (SINADEF) are in Spanish. We used death registers for 25 Peruvian regions from 1st of January through to the 31st of December (1–52 epidemiological weeks) and the preceding 3 years (2017–2019). SINADEF is a computer application. It registers data on deaths, generates death certificates, and creates a

For peer review only - http://bmjopen.bmj.com/site/about/guidelines.xhtml

statistical report; it includes fetal deaths and the deaths of unidentified persons.[17] Open access data includes information stratified by age/sex, the basic cause of death by the International Classification of Diseases 10th Revision (ICD-10), the year, and the geographic distribution. Other information is not available on SINADEF. Information on all-cause deaths where the underlying cause was known was available, with information on region, age group (0–29, 30–39, 40–49, 50–59, 60–69, 70–79, and 80 and over), and sex included. Records where the place of residence, age, sex, or year were missing were excluded.

Patient and public involvement

We used publicly available death statistics; therefore, there was no direct patient or public involvement.

Ethics statements

This study does not involve human participants. Weekly death data were obtained from SINADEF. On SINADEF, all open access data are fully anonymous and are published as part of routine surveillance (https://www.datosabiertos.gob.pe/dataset/informaci%C3%B3n-de-fallecidos-del-sistema-inform%C3%A1tico-nacional-de-defunciones-sinadef-ministerio). Therefore, this study was exempt from review by an ethics board.

Statistical analysis

We estimated the excess deaths and the excess death rates (per 100,000 inhabitants) according to sex and age, as well as in each region by sex. Expected deaths in 2020 were obtained from the average number of deaths over the years 2017–2019. The weekly average number of deaths by sex and age in the three years preceding the pandemic was the expected number of deaths. Observed deaths in 2020 were the deaths reported from 1st of January through to the 31st of December. Excess all-cause deaths during the pandemic period were estimated as the difference between observed deaths and expected deaths in 2020. To make comparisons with other countries, we measured excess proportional mortality (proportion of mortality count excess; weekly or monthly deaths in 2020) as the percentage difference between the observed and expected number of deaths ((observed deaths–expected deaths)/expected deaths*100%),[8] by sex, age, region, and epidemiological week. Mortality rates (MR) per 100,000 inhabitants for both sexes were also calculated by dividing the number of deaths per region, standardized by the estimated population of each region. We calculated the expected MR for the year 2020 and the MR observed in 2020 for both sexes according to age and region (per 100,000 inhabitants). The excess death rate was calculated using the difference between the MR observed in 2020 and the MR expected in 2020 (per 100,000 inhabitants). Population counts used for calculating the mortality rates were obtained from projections from Peru's National Institute of Statistics and Informatic (INEI, in Spanish).[18] In addition, odds ratios (ORs) with 95% confidence intervals (CIs) for observed mortality vs. expected mortality according to sex were estimated separately for each age stratum and region. These results were summarized in a random-effects meta-analysis with individual reports weighted using the weights indicated. The Mantel–Haenszel method was used to calculate the random effects estimates. $P<0.05$ was considered statistically significant. The meta-analysis was performed in RevMan 5, Cochrane.

Results

From the 1st of January through to the 31st of December 2020, a total of 553,348 all-cause deaths occurred in Peru. Of these deaths, we included a total of 302,177 and 236,733 deaths in men and women, respectively (Figure 1).

Excess death and excess death rates by sex and age

For peer review only - http://bmjopen.bmj.com/site/about/guidelines.xhtml

Between January 1 2020 and December 31 2020, a total of 127,000 all-cause deaths (MR 784 deaths per 100,000 men) were reported in men. In comparison with the expected 58,392 deaths (MR 361 per 100,000 men), the number of excess deaths during the same period was 68,608 (117%), and the excess death rate was 424 deaths per 100,000 men. During the same period, a total of 85,240 (MR 519 deaths per 100,000 women) all-cause deaths were reported in women. In comparison with the expected 50,498 deaths (MR 307 deaths per 100,000 women), the number of excess deaths during the same period was 34,742 (69%), and the excess death rate was 211 deaths per 100,000 women.

The number of excess deaths increased with age and was higher in men aged 60–69 years (217%) compared with women (121%) (Figure 2A). The all-cause mortality rate in relation to the expected death rate increased with age and was higher in individuals aged 80 years or over in both sexes (Figure 2B). In addition, the increase in the all-cause mortality rate was more than twice as high in men compared with that in women.

ORs for excess death rates stratified by sex and age

In Figure 3, our meta-analysis shows a continuous age-dependent increase in the number of excess deaths in men and women. Overall, men and women had 2.08 (95% CI, 1.59–2.73) and 1.67 (95% CI, 1.41–1.96) times increased odds of excess mortality compared to the expected mortality, respectively. Men in the 40–79 years age group had two times higher odds of excess mortality. Men in the 60–69 years age group had 3.23 (95% CI, 3.15-3.31) times increased odds of excess mortality, while women had 2.23 (95% CI, 2.16-2.29) times increased odds (Figures 3A, B).

Excess death and excess death rates by sex and regions

The excess all-cause death rate relative to the expected death rate varied between regions. For men, the excess death rate varied from 34% in Cusco to 199% in Lima, while for women it varied from 7% in Tacna and 125% in Lima. The five regions with the highest excess death rates in men were Lima (199%), Callao (143%), Piura (137%), Moquegua (129%), and Arequipa (123%); the five regions with the highest excess deaths in women were Lima (125%), Callao (94%), Paso (91%), Tumbes (83%), and Piura (78%) (Figure 4A). The highest excess death rates in men (per 100,000 men) and women (per 100,000 women) occurred in Lima, Callao, Moquegua, Arequipa, Ica, and Tumbes (Figure 4B).

ORs for excess death rates stratified by sex and regions

In Figure 5, our meta-analysis shows a continuous increase in the rate of excess deaths in men and women. Men in Lima had 3.01 (95% CI, 2.96–3.06) times increased odds of excess mortality. Men in Callao, Piura, Arequipa, Loreto, Tumbes, and Moquegua had two times increased odds of excess mortality compared to the expected mortality (Figure 5A), while only in Lima did women have 2.26 (95% CI, 2.21–2.30) times increased odds of excess mortality compared to the expected mortality (Figure 5B).

Temporal distribution of excess deaths by sex and region

For peer review only - http://bmjopen.bmj.com/site/about/guidelines.xhtml

Figure 6 shows that the rate of excess deaths increased at the beginning of March and reached a peak at the end of the months of May and August. There were different peaks among different regions. Some regions showed staggering increases, with rates of excess deaths reaching 500% in men in Loreto in week 18. The rate of excess deaths decreased by week 52 (Figure 7).

Discussion

Main findings

During the COVID-19 pandemic, Peru experienced one of the highest excess all-cause mortality rates in the world, along with Bulgaria, North Macedonia, and Serbia.[19] Excess all-cause mortality is recognized as a robust and comparable indicator of mortality associated with the COVID-19 pandemic.[20] Some countries have reported that approximately two thirds of their excess deaths were reported as COVID-19-related deaths.[21] However, in countries such as Peru, where the effects of the pandemic have exceeded the reporting systems for mortality related to COVID-19, the measurement of excess mortality can help guide public health decisions and actions.

Potential explanations and implications

According to our analysis, men in Peru had an excess mortality rate almost double that of women. This differs from the data reported in most industrialized and high-income countries, where no great difference is evident. For example, the excess mortality in Peruvian men (117%) was double that reported for England and Wales (63%), which, together with Spain, were the countries that experienced the highest excess mortality in Europe;[20,22] this is also higher than the rate reported in other countries of the American continent, such as Mexico (51%) and Brazil (61%).[8,23] Excess mortality in men is likely related to the COVID-19 pandemic, as SARS-CoV-2 has a greater transmissibility in men, which is mainly due to their social and cultural behaviors. It has also been proposed that women have a better protective factor immune response due to their higher number of B lymphocytes and the higher intensity of their inflammatory response against viral diseases.[24,25] Because COVID-19 is a new infectious disease, the development and viability of memory T cells in men and women are still unknown, especially in the face of viral mutation. Therefore, further studies could define the sex-differential role of T cells in acute disease. Sex differences in immunopathogenesis could inform mechanisms of COVID-19, and identify points for treatment and increase vaccine efficacy to target parts of the virus that are less likely to mutate, supported by genome analysis.[26,27]

Several previous studies have reported age specific excess deaths. In these studies, there is a wide variability in the estimation of excess mortality by sex and age.[8,28,29] As in Peru, other countries have also reported that excess in observed mortality amongst younger age groups was similar to or lower than expected levels.[28] This is due to the lower number of unintentional injuries occurring due to COVID-19 containment measures, such as occupational or automobile accidents, the latter being the main cause of years of life lost due to premature death.[30,31] In our study, the excess deaths (%) varied markedly in the age groups of 50–59, 60–69, and 70–79 years. The rate of excess deaths also varied with age, and was higher in men aged 60–69, 70–79, and ≥80 years, in contrast to other European countries such as Belgium, the Czech Republic, Hungary, Poland, and Scotland, where the excess death rates were higher in men of 55–64 years.[28] As in Peru, in the overall US population the excess deaths in individuals of 65 years and over were higher.[29] In our study, the ORs for excess mortality occurred in a wider range of age

For peer review only - http://bmjopen.bmj.com/site/about/guidelines.xhtml

groups for men (40–79 years old); this may be related to the high prevalence of chronic diseases in adult men, which increases the likelihood of death due to COVID-19.[32,33] Thus, a recent study showed that as obesity prevalence increases, the COVID-19 mortality rates increase in the Peruvian population ≥15 years.[34] These factors may have contributed to a higher estimated excess all-cause deaths rate in men than women in the ≥60 year age group in Peru.

Overall, there were different peaks among different regions of excess mortality. However, men were found to be twice as likely to die in more regions than women. Regions along the coast (Lima, Callao, Piura, and Tumbes), in the Andes (Arequipa and Moquegua), and in the Amazon (Loreto) have large cities with high levels of commercial, migratory, and tourist exchange. In these regions with large cities, the rates of excess death in men increased at the beginning of March, reached a peak in weeks 18 and 32, and then decreased. This increase in all-cause deaths in these regions was temporal, and concomitant with the first wave of the COVID-19 pandemic in Peru. The temporal distribution was similar in women (Figures 7A, B). In contrast, the regions of the Andes located at higher altitudes had lower mortality rates. This has been related to the physiological adaptation of this population to a hypoxic environment, which may have protected them from severe impacts of the acute infection caused by SARS-CoV-2; in addition, these areas have a lower prevalence of diabetes, obesity, and hypertension.[35] Therefore, the excess all-cause deaths are likely to be a direct effect of the COVID-19 pandemic. These characteristics, together with low compliance with social distancing measures, the closure of economic activities during the pandemic, the introduction of new variants of SARS-CoV-2 with greater transmissibility, the difficulty of early diagnosis, the collapse of hospitals, and the shortage of medical oxygen could explain the higher excess mortality in Peru.[36,37] The changes in population (male and female) behavior brought about by lockdown measures could also have had effects on excess deaths and the mortality rate. Finally, during the country-wide lockdown implemented in Peru, access to medical and surgical care was limited and interrupted across primary and secondary prevention programs. Further studies could evaluate the indirect effects of confinement and excess deaths and mortality by disaggregating data by chronic or acute diseases.

Weaknesses of this study

Our study has several important limitations. First, although SINADEF is an official database, there may have been delays in its registration due to a lack of notification of non-hospital or manually registered deaths. Therefore, the number of registered excess all-cause deaths might have been underestimated.

A second limitation is related to the selection of expected deaths; a study of excess mortality during the pandemic carried out in 103 countries mentions that 20 of them, in which Peru is located, have a death record of less than 90% according to the Demographic Yearbook of the United Nations for 2019,[19] although there was an increasing trend in the coverage of registered deaths according to data from 2012 (70%) to 2016 (80%). In 2017, improvements to the SINADEF of Peru—with the collaboration of international institutions that allowed the increase in the use of death certificates online and the improvement in the quality of information through training, standardization, and monitoring of civil registration and vital statistics processes—shortened the gap between coverage of registered and estimated deaths.[38] However, it is likely that an increase in the registration of deaths expected during 2017–2019 is related to coverage of deaths; therefore, the use of an average calculated in epidemiological weeks would be justified.

Third, the analysis could limit its comparison with other studies, where excess mortality uses complex models that apply periodic splines or Fourier harmonics to smooth and homogenize possible fluctuations in expected deaths and standardize their measurements.[39] We

For peer review only - http://bmjopen.bmj.com/site/about/guidelines.xhtml

used the proportion of mortality count excess and a standard measure (P-score), which is simple and easy to replicate in any context and similar to that carried out in other studies.[8,29] Moreover, while other studies estimated the expected deaths as the average number of deaths between 2015–2019, we only used the three years preceding the pandemic (2017–2019). This method could also be easily replicated in each region without losing the opportunity to analyze important variables;[8] however, it was only possible to access three years as a baseline. Future analysis of excess deaths in 2021 could not include 2020 in the average expected deaths due to high death rates.

Strengths of this study

The main strength of our study is that the developed method allows for transparency and reproducibility of the findings. The baseline for expected deaths made it possible to capture the weekly variation of observed deaths and is self-consistent with that reported in more complex methodologies.[39] The simplicity in the analysis of excess mortality allows for the opportunity to use the findings in epidemiological surveillance and their interpretation by those responsible for the formulation of public policies and health authorities at different levels.

Implications for policymakers

The findings add to the body of evidence about our understanding of excess mortality in Peru. Although the initiation of vaccinations against COVID-19 and improvements in the capacity to diagnose and care for critical patients will reduce mortality, policymakers must evaluate future measures and interventions based on gender. On the other hand, coordination initiatives between those responsible for epidemiological surveillance, vital event registration systems, and diagnosis have contributed to obtaining more realistic numbers, but evaluating the interoperability of computer and statistical systems is necessary for future measurements of observed deaths and excess mortality. Finally, having up-to-date information provides government and public health officials with adequate mechanisms to track the impact of the pandemic.

Conclusion

Approximately one hundred thousand excess deaths occurred in men and women during the COVID-19 pandemic in Peru. The age-stratified excess death rates were higher in men than in women, and men between the ages of 40 and 79 years experienced twice the rate of excess deaths compared with the rate expected. Strong excesses in geographical and temporal mortality patterns by region were found in both men and women. These findings reveal the impact of the COVID-19 pandemic on all-cause mortality up to the point where vaccination against SARS-CoV-2 started to become available in Peru.

Footnotes

Contributors. MCRS designed the study and oversaw the acquisition of data. MCRS, GOC, and HAH performed the data collection and interpretation of the data. MCRS and HAH verified the coded results. MCRS and HAH analyzed the data. MCRS, GOC, and HAH contributed to drafting the manuscript. MCRS, GOC, and HAH were involved in critical revisions of the manuscript for important intellectual content. All authors approved the final draft of the manuscript.

Funding. This study has no funding.

For peer review only - http://bmjopen.bmj.com/site/about/guidelines.xhtml

Competing. The authors declare no conflict of interest.

Ethics statements. Patient consent for publication was not required.

Data availability statement. Data are available in a public, open access repository. Data used are freely available and can be accessed from Peruvian Ministry of Health (National Information System of Deaths), and National Institute of Statistics and Informatics.

Acknowledgments. Not available.

References

1. Dhama K, Khan S, Tiwari R, Sircar S, Bhat S, Malik YS, et al. Coronavirus Disease 2019-COVID-19. Clin Microbiol Rev. 2020;33(4):e00028-20.
2. O'Driscoll M, Ribeiro Dos Santos G, Wang L, Cummings DAT, Azman AS, Paireau J, et al. Age-specific mortality and immunity patterns of SARS-CoV-2. Nature. 2021;590(7844):140-145.
3. Bilinski A, Emanuel EJ. COVID-19 and Excess All-Cause Mortality in the US and 18 Comparison Countries. JAMA. 2020;324(20):2100-2102.
4. Chen YH, Glymour MM, Catalano R, Fernandez A, Nguyen T, Kushel M, et al. Excess Mortality in California During the Coronavirus Disease 2019 Pandemic, March to August 2020. JAMA Intern Med. 2021 May 1;181(5):705-707.
5. Banerjee A, Pasea L, Harris S, Gonzalez-Izquierdo A, Torralbo A, Shallcross L, et al. Estimating excess 1-year mortality associated with the COVID-19 pandemic according to underlying conditions and age: a population-based cohort study. Lancet. 2020;395(10238):1715-1725.
6. Kontopantelis E, Mamas MA, Deanfield J, Asaria M, Doran T. Excess mortality in England and Wales during the first wave of the COVID-19 pandemic. J Epidemiol Community Health. 2021;75(3):213-223.
7. Gianicolo EAL, Russo A, Büchler B, Taylor K, Stang A, Blettner M. Gender specific excess mortality in Italy during the COVID-19 pandemic accounting for age. Eur J Epidemiol. 2021 Feb;36(2):213-218.
8. Palacio-Mejía LS, Wheatley-Fernández JL, Ordóñez-Hernández I, López-Ridaura R, López Gatell-Ramírez H, Hernández-Ávila M, et al. All-cause excess mortality during the Covid-19 pandemic in Mexico. Salud Publica Mex. 2021;63:211-224
9. Locatelli I, Rousson V. A first analysis of excess mortality in Switzerland in 2020. PLoS ONE, 2021;16(6): e0253505
10. Liu J, Zhang L, Yan Y, Zhou Y, Yin P, Qi J, et al. Excess mortality in Wuhan city and other parts of China during the three months of the covid-19 outbreak: findings from nationwide mortality registries. BMJ. 2021;372:n415.
11. Islam N, Shkolnikov VM, Acosta RJ, Klimkin I, Kawachi I, Irizarry RA, et al. Excess deaths associated with covid-19 pandemic in 2020: age and sex disaggregated time series analysis in 29 high income countries. BMJ. 2021;373:n1137.
12. Peruvian Ministry of Health (MINSA). Sala Situacional. COVID-19 Peru. Lima: MINSA; 2021. Available: https://covid19.minsa.gob.pe/ [accessed on 27 Julay 2021]
13. Peruvian Ministry of Health (MINSA). Excess Mortality. Lima: MINSA; 2021. Available: https://www.minsa.gob.pe/reunis/data/exceso_mortalidad.asp [accessed on 03 June 2021]
14. Ramírez-Soto MC, Arroyo-Hernández H, Ortega-Cáceres G. Sex differences in the incidence, mortality, and fatality of COVID-19 in Peru. PLoS ONE. 2021;16(6): e0253193.

For peer review only - http://bmjopen.bmj.com/site/about/guidelines.xhtml

15. Munayco C, Chowell G, Tariq A, Undurraga EA, Mizumoto K. Risk of death by age and gender from CoVID-19 in Peru, March-May, 2020. Aging (Albany NY). 2020;12(14):13869–81.
16. Vandenbroucke JP, von Elm E, Altman DG, Gøtzsche PC, Mulrow CD, Pocock SJ, Poole C, Schlesselman JJ, Egger M; STROBE Initiative. Strengthening the Reporting of Observational Studies in Epidemiology (STROBE): explanation and elaboration. PLoS Med. 2007 Oct 16;4(10):e297.
[dataset] 17. Peruvian Ministry of Health (MINSA). Data from: National Information System of Deaths (SINADEF). MINSA Digital Repository, June 03, 2021. https://www.datosabiertos.gob.pe/dataset/informaci%C3%B3n-de-fallecidos-del-sistema-inform%C3%A1tico-nacional-de-defunciones-sinadef-ministerio
[dataset] 18. National Institute of Statistics and Informatics (INEI). Data from: Population estimates and projections, 2015–2021. INEI Digital Repository, June 03, 2021. https://www.inei.gob.pe/estadisticas/indice-tematico/population-estimates-and-projections/
19. Karlinsky A, Kobak D. Tracking excess mortality across countries during the COVID-19 pandemic with the World Mortality Dataset. Elife. 2021;10:e69336.
20. Vestergaard LS, Mølbak K. Timely monitoring of total mortality associated with COVID-19: informing public health and the public. Euro Surveill. 2020;25(34):2001591.
21. Woolf SH, Chapman DA, Sabo RT, Weinberger DM, Hill L, Taylor DDH. Excess Deaths From COVID-19 and Other Causes, March-July 2020. JAMA 2020;324:1562-4. Doi:10.1001/jama.2020.19545
22. Kontis V, Bennett JE, Rashid T, Parks RM, Pearson-Stuttard J, Guillot M, *et al.* Magnitude, demographics and dynamics of the effect of the first wave of the Covid-19 pandemic on all-cause mortality in 21 industrialized countries. Nat Med. 2020;26:1919-28.
23. Silva GAE, Jardim BC, Santos C. Excess mortality in Brazil in times of Covid-19. Cien Saude Colet. 2020;25(9):3345-54.
24. Gebhard C, Regitz-Zagrosek V, Neuhauser HK, Morgan R, Klein SL. Impact of sex and gender on COVID-19 outcomes in Europe. Biol Sex Differ. 2020; 11(1):29. 11:29. pmid:32450906
25. Abate BB, Kassie AM, Kassaw MW, Aragie TG, Masresha SA. Sex difference in coronavirus disease (COVID-19): a systematic review and meta-analysis. BMJ Open. 2020 Oct 6;10(10):e040129.
26. Scully EP, Haverfield J, Ursin RL, Tannenbaum C, Klein SL. Considering how biological sex impacts immune responses and COVID-19 outcomes. Nat Rev Immunol. 2020 Jul;20(7):442-447. doi: 10.1038/s41577-020-0348-8.
27. Gadi N, Wu SC, Spihlman AP, Moulton VR. What's Sex Got to Do With COVID-19? Gender-Based Differences in the Host Immune Response to Coronaviruses. Front Immunol. 2020 Aug 28;11:2147. doi: 10.3389/fimmu.2020.02147.
28. Islam N, Shkolnikov VM, Acosta RJ, Klimkin I, Kawachi I, Irizarry RA. Excess deaths associated with covid-19 pandemic in 2020: age and sex disaggregated time series analysis in 29 high income countries. BMJ 2021;373:n1137.
29. Aron J, Muellbauer J. A Pandemic Primer on Excess Mortality Statistics and Their Comparability Across Countries. Our World in Data; 2020. Available in: https://ourworldindata.org/covid-excess-mortality
30. Velásquez A. La carga de enfermedad y lesiones en el Perú y las prioridades del plan esencial de aseguramiento universal. Rev Perú Med Exp Salud Publica. 2009; 26(2): 222-231.
31. Calderon-Anyosa RJC, Kaufman JS. Impact of COVID-19 lockdown policy on homicide, suicide, and motor vehicle deaths in Peru. Prev Med. 2021;143:106331

For peer review only - http://bmjopen.bmj.com/site/about/guidelines.xhtml

32. Yamada G, Castillo-Salgado C, Jones-Smith JC, Moulton LH. Differences in magnitude and rate of change in adult obesity distribution by age and sex in Mexico, Colombia and Peru, 2005-2010. Public Health Nutr. 2019 Mar;22(4):757-763.

33. Zhou F, Yu T, Du R, et al. Clinical course and risk factors for mortality of adult inpatients with COVID-19 in Wuhan, China: a retrospective cohort study. Lancet. 2020;395(10229):1054–1062.

34. Ramírez-Soto MC, Alarcón-Arroyo M, Chilcon-Vitor Y, Chirinos-Pérez Y, Quispe-Vargas G, Solsol-Jacome K, et al. Association between Obesity and COVID-19 Mortality in Peru: An Ecological Study. Tropical Medicine and Infectious Disease. 2021;6(4):182.

35. Seclén SN, Nunez-Robles E, Yovera-Aldana M, Arias-Chumpitaz A. Incidence of COVID-19 infection and prevalence of diabetes, obesity and hypertension according to altitude in Peruvian population. Diabetes Res Clin Pract. 2020 Nov;169:108463.

36. Álvarez-Antonio C, Meza-Sánchez G, Calampa C, Casanova W, Carey C, Alava F. et al. Seroprevalence of anti-SARS-CoV-2 antibodies in Iquitos, Peru in July and August, 2020: a population-based study. Lancet Glob Health. 2021 Jul;9(7):e925-e931.

37. Reyes-Vega MF, Soto-Cabezas MG, Cárdenas F, Martel KS, Valle A, Valverde J. et al. SARS-CoV-2 prevalence associated to low socioeconomic status and overcrowding in an LMIC megacity: A population-based seroepidemiological survey in Lima, Peru. EClinicalMedicine. 2021;34:100801.

38. Vargas-Herrera J, Pardo-Ruiz K, Garro-Nuñez G, Miki-Ohno J, Pérez-Lu JE, Valdez-Huarcaya W, et al. [Preliminary results of the strengthening of the national death registry information system]. Rev Peru Med Exp Salud Publica. 2018;35(3):505–514. (in Spanish)

39. Sempé L, Lloyd-Sherlock P, Martínez R, Ebrahim S, McKee M, Acosta E. Estimation of all-cause excess mortality by age-specific mortality patterns for countries with incomplete vital statistics: a population-based study of the case of Peru during the first wave of the COVID-19 pandemic. Lancet Reg Health Am. 2021.

Legends:

Figure 1. The study flow chart of all-cause death excess using the STROBE (Strengthening the Reporting of Observational Studies in Epidemiology) reporting guidelines.

Figure 2. Excess numbers of (A) deaths and (B) excess death rates in men and women in Peru stratified by age.

Figure 3. ORs for excess death rates stratified by (A) age in men and (B) women in Peru. ORs with 95% confidence intervals (CIs) estimated by age and sex for observed mortality in 2020 vs. expected mortality in 2020 (average number of deaths over the years 2017–2019).

Figure 4. (A) Observed, expected, and excess death and (B) excess death rates stratified by region in men and women in Peru.

Figure 5. ORs for excess death rates according to region in (A) men and (B) women in Peru. ORs with 95% confidence intervals (CIs) estimated by region and sex for observed mortality in 2020 vs. expected mortality in 2020 (average number of deaths over the years 2017–2019).

Figure 6. Temporal distribution of excess deaths (%) in men and women in Peru.

For peer review only - http://bmjopen.bmj.com/site/about/guidelines.xhtml

Figure 7. Temporal distribution of excess deaths according to region in (A) men and (B) women in Peru.

For peer review only

For peer review only - http://bmjopen.bmj.com/site/about/guidelines.xhtml

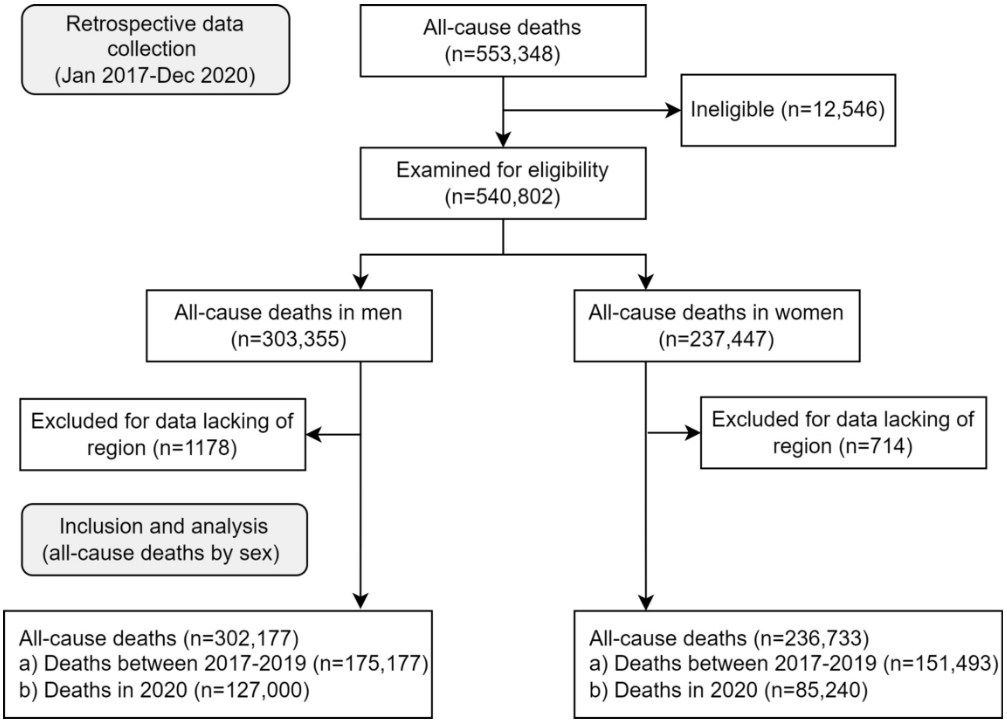

Figure 1. The study flow chart of all-cause death excess using the STROBE (Strengthening the Reporting of Observational Studies in Epidemiology) reporting guidelines.

190x136mm (300 x 300 DPI)

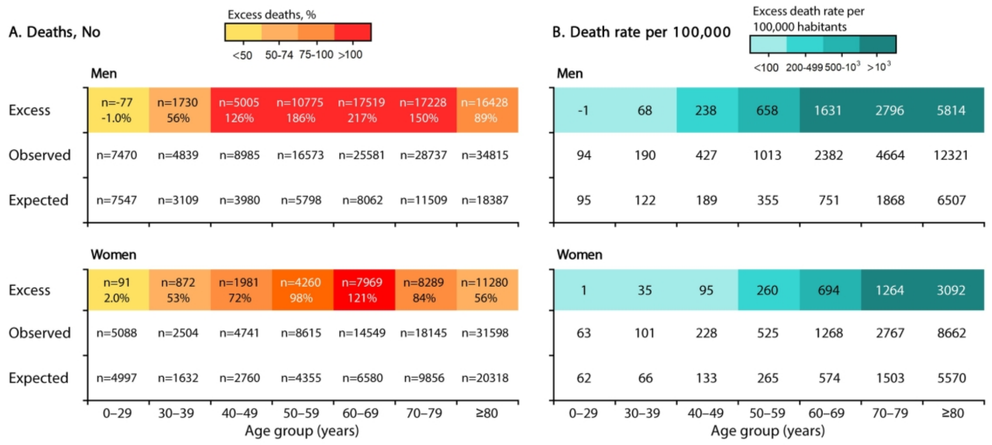

Figure 2. Excess numbers of (A) deaths and (B) excess death rates in men and women in Peru stratified by age.

190x86mm (300 x 300 DPI)

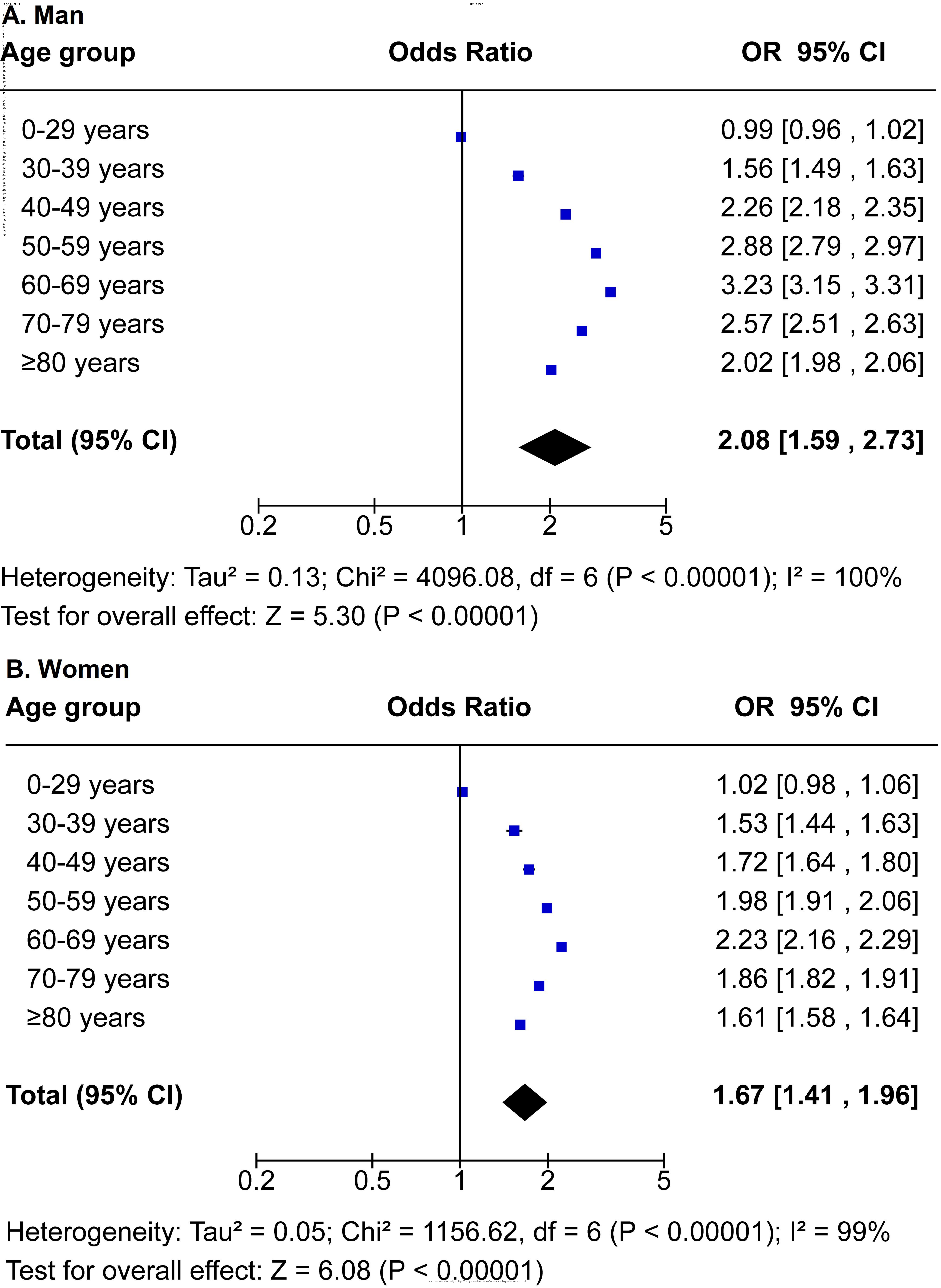
A. Man
Age group
Odds Ratio
OR 95% CI
0-29 years
0.99 [0.96 , 1.02]
30-39 years
1.56 [1.49 , 1.63]
40-49 years
2.26 [2.18 , 2.35]
50-59 years
2.88 [2.79 , 2.97]
60-69 years
3.23 [3.15 , 3.31]
70-79 years
2.57 [2.51 , 2.63]
≥80 years
2.02 [1.98 , 2.06]
Total (95% CI)
2.08 [1.59 , 2.73]
0.2
0.5
1
2
5
Heterogeneity: Tau² = 0.13; Chi² = 4096.08, df = 6 (P < 0.00001); I² = 100%
Test for overall effect: Z = 5.30 (P < 0.00001)
B. Women
Age group
Odds Ratio
OR 95% CI
0-29 years
1.02 [0.98 , 1.06]
30-39 years
1.53 [1.44 , 1.63]
40-49 years
1.72 [1.64 , 1.80]
50-59 years
1.98 [1.91 , 2.06]
60-69 years
2.23 [2.16 , 2.29]
70-79 years
1.86 [1.82 , 1.91]
≥80 years
1.61 [1.58 , 1.64]
Total (95% CI)
1.67 [1.41 , 1.96]
0.2
0.5
1
2
5
Heterogeneity: Tau² = 0.05; Chi² = 1156.62, df = 6 (P < 0.00001); I² = 99%
Test for overall effect: Z = 6.08 (P < 0.00001)

A. Deaths

Excess deaths, %: <50; 51-75; 76-99; ≥100

	Men			Women		
	Expected	Observed	Excess	Expected	Observed	Excess
Lima	17273	51668	34395 (199%)	14986	33683	18697 (125%)
Callao	2453	5951	3498 (143%)	1936	3752	1816 (94%)
Pasco	340	675	335 (98%)	282	539	257 (91%)
Tumbes	511	1073	562 (110%)	384	704	320 (83%)
Piura	2921	6921	4000 (137%)	2463	4393	1930 (78%)
Loreto	1250	2769	1519 (122%)	947	1678	731 (77%)
Arequipa	3088	6897	3809 (123%)	2665	4633	1968 (74%)
Ucayali	997	1852	855 (86%)	726	1196	470 (65%)
Madre de Dios	379	729	350 (93%)	213	349	136 (64%)
Ica	2226	4651	2425 (109%)	1992	3114	1122 (56%)
Moquegua	427	977	550 (129%)	354	552	198 (56%)
San Martin	1495	2591	1096 (73%)	1124	1621	497 (44%)
Apurimac	805	1151	346 (43%)	724	1041	317 (44%)
La Libertad	4103	7433	3330 (81%)	3664	5181	1517 (41%)
Junin	2968	4888	1920 (65%)	2566	3617	1051 (41%)
Ancash	2786	5058	2272 (82%)	2517	3519	1002 (40%)
Cajamarca	1655	2943	1288 (78%)	1576	2152	576 (37%)
Huanuco	1545	2240	695 (45%)	1412	1798	386 (27%)
Ayacucho	903	1409	506 (56%)	882	1116	234 (27%)
Huancavelica	937	1297	360 (38%)	928	1129	201 (22%)
Cusco	3212	4317	1105 (34%)	2747	3341	594 (22%)
Puno	2761	3988	1227 (44%)	2521	3028	507 (20%)
Amazonas	477	681	204 (43%)	396	438	42 (11%)
Tacna	719	1086	367 (51%)	626	668	42 (7%)
Lambayeque	1865	3439	1574 (84%)	1691	1801	110 (7%)

B. Mortality

Mortality Rate per 100,000 hab.: <100; 100-199; 200-299; ≥300

	Men				Women			
		Expected	Observed	Excess		Expected	Observed	Excess
Lima		337	1009	672	Lima	272	611	339
Callao		446	1082	636	Callao	334	647	313
Moquegua		415	950	535	Moquegua	393	614	221
Arequipa		420	937	517	Arequipa	350	608	258
Ica		455	951	496	Ica	410	640	230
Tumbes		377	791	414	Tumbes	332	608	276
Piura		283	671	388	Piura	242	432	190
Ancash		468	850	382	Ancash	430	601	171
Madre de Dios		386	742	356	Madre de Dios	282	462	180
La Libertad		410	743	333	La Libertad	360	510	150
Loreto		235	521	286	Loreto	191	338	147
Junin		437	720	283	Junin	376	530	154
Ucayali		324	602	278	Ucayali	258	425	167
Pasco		243	481	238	Pasco	214	409	195
Lambayeque		292	539	247	Lambayeque	251	268	17
San Martin		315	546	231	San Martin	264	381	117
Huancavelica		509	704	195	Huancavelica	512	623	111
Puno		451	652	201	Puno	402	483	81
Huanuco		402	583	181	Huanuco	376	478	102
Tacna		382	577	195	Tacna	342	365	23
Cajamarca		228	405	177	Cajamarca	217	296	79
Apurimac		365	522	157	Apurimac	344	495	151
Cusco		468	629	161	Cusco	410	498	88
Ayacucho		264	412	148	Ayacucho	270	342	72
Amazonas		217	310	93	Amazonas	191	212	21

Figure 4. (A) Observed, expected, and excess death and (B) excess death rates stratified by region in men and women in Peru.

181x216mm (400 x 400 DPI)

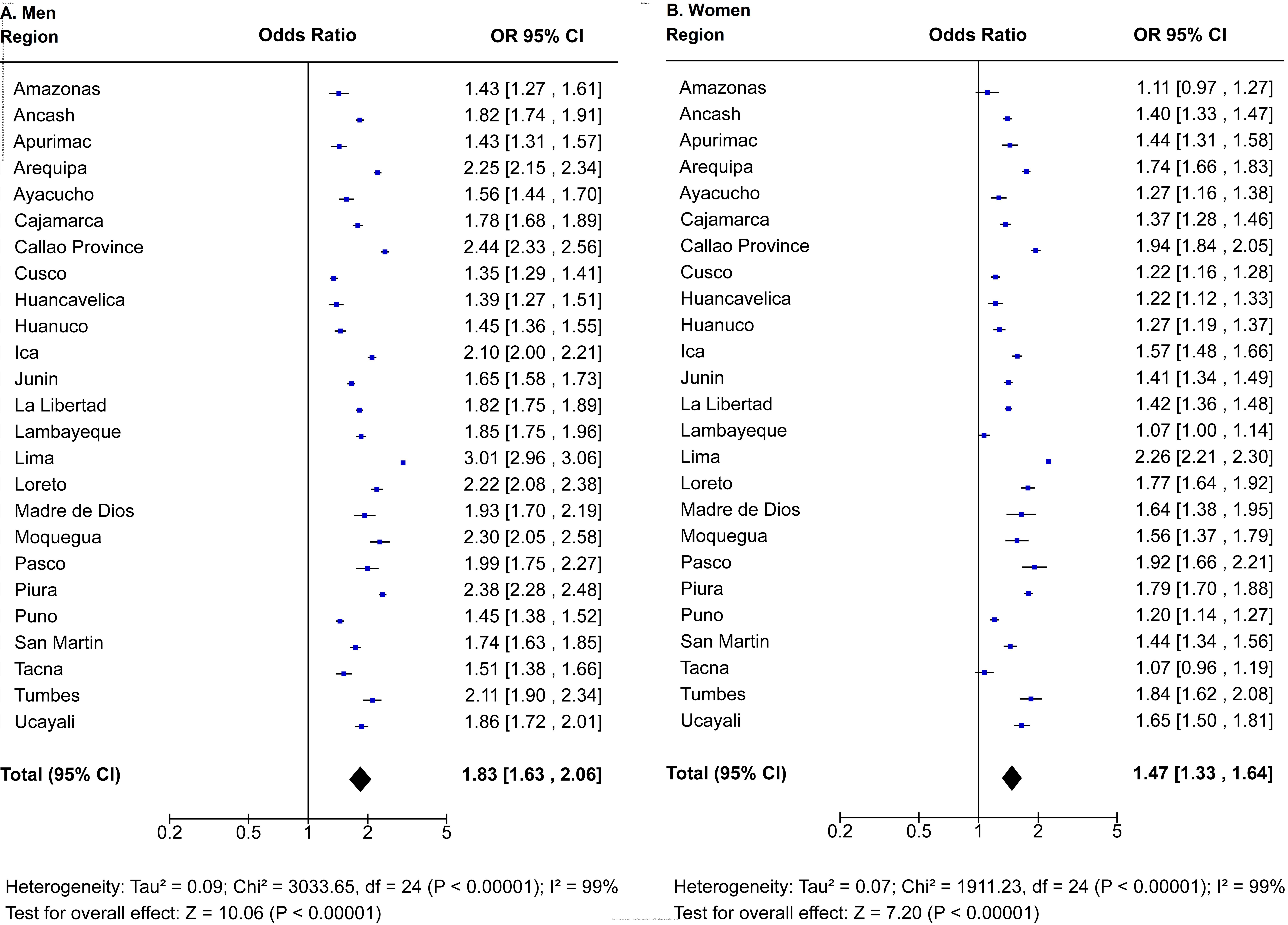
A. Men
Region
Odds Ratio
OR 95% CI
Amazonas 1.43 [1.27 , 1.61]
Ancash 1.82 [1.74 , 1.91]
Apurimac 1.43 [1.31 , 1.57]
Arequipa 2.25 [2.15 , 2.34]
Ayacucho 1.56 [1.44 , 1.70]
Cajamarca 1.78 [1.68 , 1.89]
Callao Province 2.44 [2.33 , 2.56]
Cusco 1.35 [1.29 , 1.41]
Huancavelica 1.39 [1.27 , 1.51]
Huanuco 1.45 [1.36 , 1.55]
Ica 2.10 [2.00 , 2.21]
Junin 1.65 [1.58 , 1.73]
La Libertad 1.82 [1.75 , 1.89]
Lambayeque 1.85 [1.75 , 1.96]
Lima 3.01 [2.96 , 3.06]
Loreto 2.22 [2.08 , 2.38]
Madre de Dios 1.93 [1.70 , 2.19]
Moquegua 2.30 [2.05 , 2.58]
Pasco 1.99 [1.75 , 2.27]
Piura 2.38 [2.28 , 2.48]
Puno 1.45 [1.38 , 1.52]
San Martin 1.74 [1.63 , 1.85]
Tacna 1.51 [1.38 , 1.66]
Tumbes 2.11 [1.90 , 2.34]
Ucayali 1.86 [1.72 , 2.01]
Total (95% CI) 1.83 [1.63 , 2.06]
0.2
0.5
1
2
5
Heterogeneity: Tau² = 0.09; Chi² = 3033.65, df = 24 (P < 0.00001); I² = 99%
Test for overall effect: Z = 10.06 (P < 0.00001)
B. Women
Region
Odds Ratio
OR 95% CI
Amazonas 1.11 [0.97 , 1.27]
Ancash 1.40 [1.33 , 1.47]
Apurimac 1.44 [1.31 , 1.58]
Arequipa 1.74 [1.66 , 1.83]
Ayacucho 1.27 [1.16 , 1.38]
Cajamarca 1.37 [1.28 , 1.46]
Callao Province 1.94 [1.84 , 2.05]
Cusco 1.22 [1.16 , 1.28]
Huancavelica 1.22 [1.12 , 1.33]
Huanuco 1.27 [1.19 , 1.37]
Ica 1.57 [1.48 , 1.66]
Junin 1.41 [1.34 , 1.49]
La Libertad 1.42 [1.36 , 1.48]
Lambayeque 1.07 [1.00 , 1.14]
Lima 2.26 [2.21 , 2.30]
Loreto 1.77 [1.64 , 1.92]
Madre de Dios 1.64 [1.38 , 1.95]
Moquegua 1.56 [1.37 , 1.79]
Pasco 1.92 [1.66 , 2.21]
Piura 1.79 [1.70 , 1.88]
Puno 1.20 [1.14 , 1.27]
San Martin 1.44 [1.34 , 1.56]
Tacna 1.07 [0.96 , 1.19]
Tumbes 1.84 [1.62 , 2.08]
Ucayali 1.65 [1.50 , 1.81]
Total (95% CI) 1.47 [1.33 , 1.64]
0.2
0.5
1
2
5
Heterogeneity: Tau² = 0.07; Chi² = 1911.23, df = 24 (P < 0.00001); I² = 99%
Test for overall effect: Z = 7.20 (P < 0.00001)

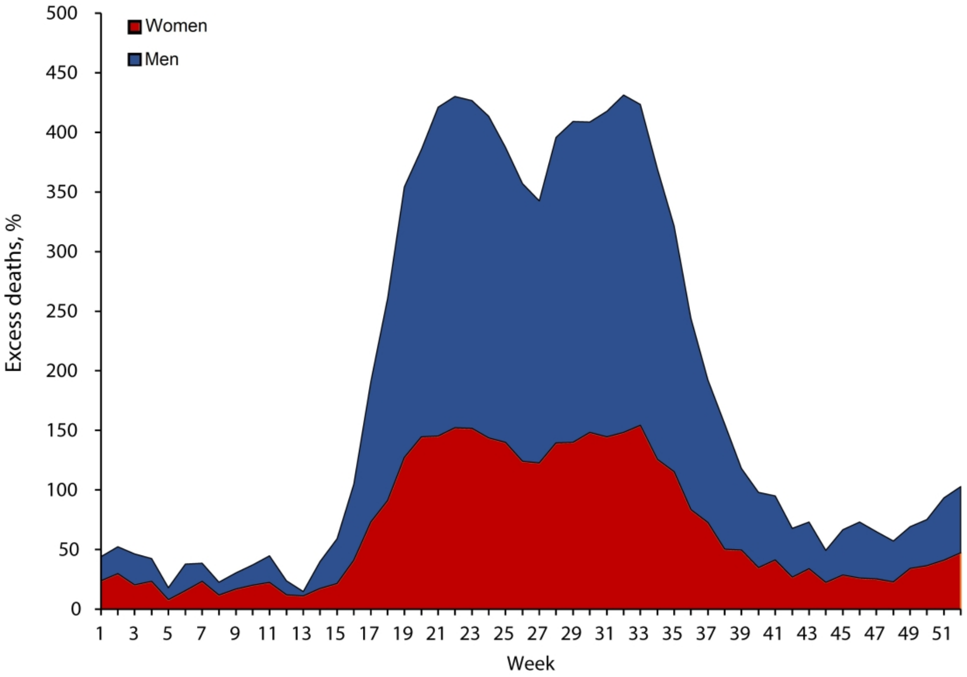

Figure 6. Temporal distribution of excess deaths (%) in men and women in Peru.

154x110mm (600 x 600 DPI)

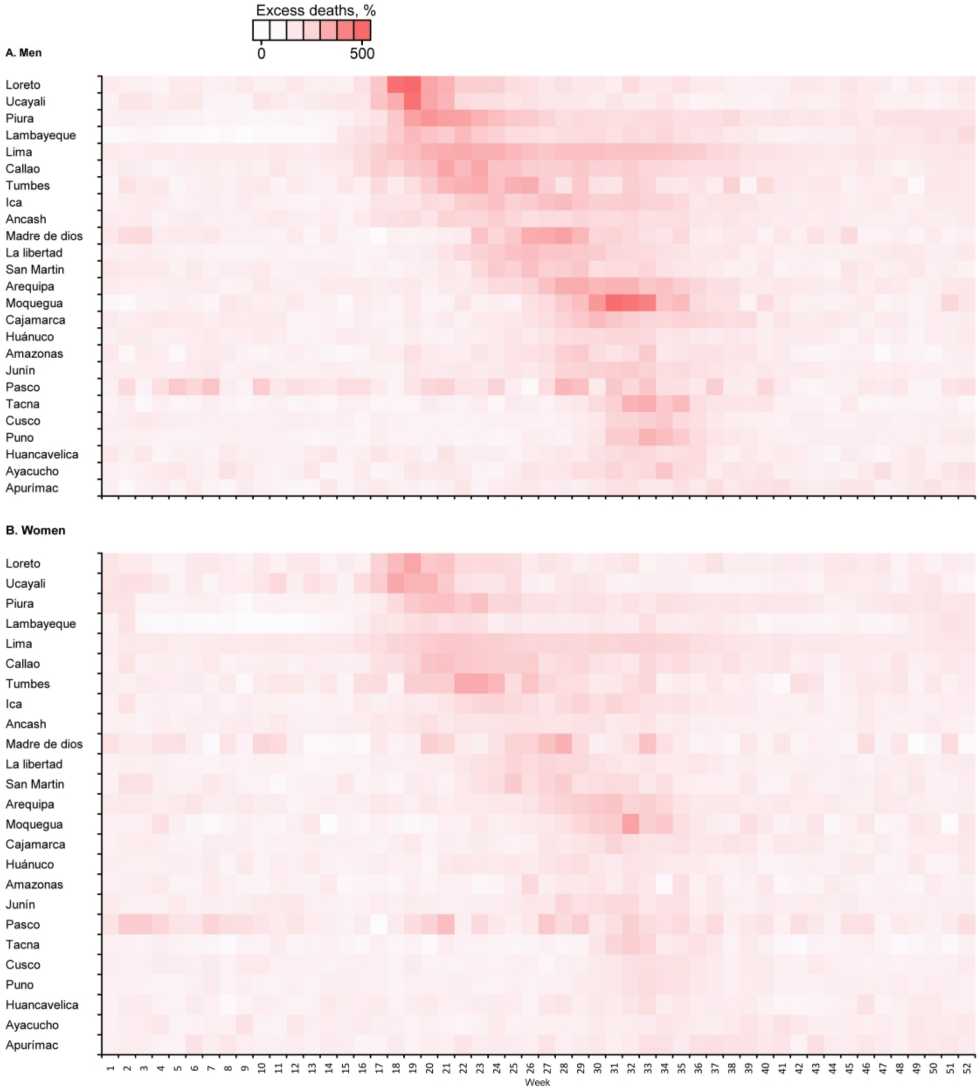

Figure 7. Temporal distribution of excess deaths according to region in (A) men and (B) women in Peru.

181x199mm (400 x 400 DPI)

STROBE Statement—checklist of items that should be included in reports of observational studies

	Item No	Recommendation	Page No
**Title and abstract**	1	(*a*) Indicate the study's design with a commonly used term in the title or the abstract	1
		(*b*) Provide in the abstract an informative and balanced summary of what was done and what was found	2
**Introduction**			
Background/rationale	2	Explain the scientific background and rationale for the investigation being reported	4
Objectives	3	State specific objectives, including any prespecified hypotheses	4
**Methods**			
Study design	4	Present key elements of study design early in the paper	4
Setting	5	Describe the setting, locations, and relevant dates, including periods of recruitment, exposure, follow-up, and data collection	5
Participants	6	(*a*) *Cohort study*—Give the eligibility criteria, and the sources and methods of selection of participants. Describe methods of follow-up<br>*Case-control study*—Give the eligibility criteria, and the sources and methods of case ascertainment and control selection. Give the rationale for the choice of cases and controls<br>*Cross-sectional study*—Give the eligibility criteria, and the sources and methods of selection of participants	Fig. 1
		(*b*) *Cohort study*—For matched studies, give matching criteria and number of exposed and unexposed<br>*Case-control study*—For matched studies, give matching criteria and the number of controls per case	NA
Variables	7	Clearly define all outcomes, exposures, predictors, potential confounders, and effect modifiers. Give diagnostic criteria, if applicable	5
Data sources/ measurement	8*	For each variable of interest, give sources of data and details of methods of assessment (measurement). Describe comparability of assessment methods if there is more than one group	*5*
Bias	9	Describe any efforts to address potential sources of bias	5
Study size	10	Explain how the study size was arrived at	5

For peer review only

Quantitative variables	11	Explain how quantitative variables were handled in the analyses. If applicable, describe which groupings were chosen and why	5
Statistical methods	12	(*a*) Describe all statistical methods, including those used to control for confounding	5
		(*b*) Describe any methods used to examine subgroups and interactions	5
		(*c*) Explain how missing data were addressed	5
		(*d*) *Cohort study*—If applicable, explain how loss to follow-up was addressed<br>*Case-control study*—If applicable, explain how matching of cases and controls was addressed<br>*Cross-sectional study*—If applicable, describe analytical methods taking account of sampling strategy	NA
		(*e*) Describe any sensitivity analyses	NA

Continued on next page

For peer review only

**Results**			
Participants	13*	(a) Report numbers of individuals at each stage of study—eg numbers potentially eligible, examined for eligibility, confirmed eligible, included in the study, completing follow-up, and analysed	5
		(b) Give reasons for non-participation at each stage	5
		(c) Consider use of a flow diagram	5
Descriptive data	14*	(a) Give characteristics of study participants (eg demographic, clinical, social) and information on exposures and potential confounders	5
		(b) Indicate number of participants with missing data for each variable of interest	5
		(c) *Cohort study*—Summarise follow-up time (eg, average and total amount)	NA
Outcome data	15*	*Cohort study*—Report numbers of outcome events or summary measures over time	*NA*
		Case-control study—Report numbers in each exposure category, or summary measures of exposure	*NA*
		Cross-sectional study—Report numbers of outcome events or summary measures	*NA*
Main results	16	(*a*) Give unadjusted estimates and, if applicable, confounder-adjusted estimates and their precision (eg, 95% confidence interval). Make clear which confounders were adjusted for and why they were included	6
		(*b*) Report category boundaries when continuous variables were categorized	6
		(*c*) If relevant, consider translating estimates of relative risk into absolute risk for a meaningful time period	6
Other analyses	17	Report other analyses done—eg analyses of subgroups and interactions, and sensitivity analyses	6
**Discussion**			
Key results	18	Summarise key results with reference to study objectives	7
Limitations	19	Discuss limitations of the study, taking into account sources of potential bias or imprecision. Discuss both direction and magnitude of any potential bias	7
Interpretation	20	Give a cautious overall interpretation of results considering objectives, limitations, multiplicity of analyses, results from similar studies, and other relevant evidence	8-9
Generalisability	21	Discuss the generalisability (external validity) of the study results	8-9
**Other information**			

For peer review only

Funding	22	Give the source of funding and the role of the funders for the present study and, if applicable, for the original study on which the present article is based	9

*Give information separately for cases and controls in case-control studies and, if applicable, for exposed and unexposed groups in cohort and cross-sectional studies.

Note: An Explanation and Elaboration article discusses each checklist item and gives methodological background and published examples of transparent reporting. The STROBE checklist is best used in conjunction with this article (freely available on the Web sites of PLoS Medicine at http://www.plosmedicine.org/, Annals of Internal Medicine at http://www.annals.org/, and Epidemiology at http://www.epidem.com/). Information on the STROBE Initiative is available at www.strobe-statement.org.
NA: Not available

For peer review only
